# Novel Starting Points for Human Glycolate Oxidase Inhibitors, Revealed by Crystallography-Based Fragment Screening

**DOI:** 10.3389/fchem.2022.844598

**Published:** 2022-05-04

**Authors:** Sabrina R. Mackinnon, Gustavo A. Bezerra, Tobias Krojer, Tamas Szommer, Frank von Delft, Paul E. Brennan, Wyatt W. Yue

**Affiliations:** ^1^ Centre for Medicines Discovery, Nuffield Department of Medicine, University of Oxford, Oxford, United Kingdom; ^2^ Target Discovery Institute, University of Oxford, Oxford, United Kingdom; ^3^ Diamond Light Source, Harwell Science and Innovation Campus, Didcot, United Kingdom

**Keywords:** glycolate oxidase, glyoxylate metabolism, primary hyperoxaluria, fragment-based drug discovery, substrate reduction therapy

## Abstract

Primary hyperoxaluria type I (PH1) is caused by AGXT gene mutations that decrease the functional activity of alanine:glyoxylate aminotransferase. A build-up of the enzyme’s substrate, glyoxylate, results in excessive deposition of calcium oxalate crystals in the renal tract, leading to debilitating renal failure. Oxidation of glycolate by glycolate oxidase (or hydroxy acid oxidase 1, HAO1) is a major cellular source of glyoxylate, and siRNA studies have shown phenotypic rescue of PH1 by the knockdown of HAO1, representing a promising inhibitor target. Here, we report the discovery and optimization of six low-molecular-weight fragments, identified by crystallography-based fragment screening, that bind to two different sites on the HAO1 structure: at the active site and an allosteric pocket above the active site. The active site fragments expand known scaffolds for substrate-mimetic inhibitors to include more chemically attractive molecules. The allosteric fragments represent the first report of non-orthosteric inhibition of any hydroxy acid oxidase and hold significant promise for improving inhibitor selectivity. The fragment hits were verified to bind and inhibit HAO1 in solution by fluorescence-based activity assay and surface plasmon resonance. Further optimization cycle by crystallography and biophysical assays have generated two hit compounds of micromolar (44 and 158 µM) potency that do not compete with the substrate and provide attractive starting points for the development of potent and selective HAO1 inhibitors.

## Introduction

Primary hyperoxalurias are inborn errors of glyoxylate metabolism in the liver (Cochat et al., 2012; Salido et al., 2012). The biochemical hallmark is over-accumulated glyoxylate, which is oxidized to oxalate and deposited as calcium oxalate crystals in renal tissues, leading to progressive renal damage. Once the kidney filtration rate is exceeded, systemic deposition of oxalate ensues causing life-threatening damage to bones, heart, and other tissues (Cochat et al., 2012). The most common and severe form of primary hyperoxaluria is type 1 (PH1, OMIM 259900) (Hopp et al., 2015) with an estimated prevalence of 1–3 per million population and an incidence of 1:100,000 live births in Europe (Cochat et al., 1995; Kopp and Leumann 1995; van Woerden et al., 2003; Milliner et al., 2017). PH1 is caused by loss-of-function mutations in the *AGXT* gene (Danpure and Jennings 1986), encoding the PLP-dependent enzyme alanine:glycine aminotransferase (AGXT, EC 2.6.1.44) that catalyzes the transamination of glyoxylate and alanine to glycine and pyruvate in the hepatocyte peroxisome.

At present, the only definitive treatment is combined liver-kidney transplantation, since the liver is the source of oxalate, and the kidney is the first organ damaged by it. Organ transplantation entails considerable risks associated with long-term immunosuppression (Danpure 2005), limited organ availability, and increased morbidity and mortality (Harambat et al., 2012). Alternative therapies are needed, such as substrate reduction therapy, aimed at mitigating the toxic accumulation of metabolite(s) due to the defect by inhibiting an enzyme upstream of it (Yue, Mackinnon, and Bezerra 2019).

In the context of PH1, glycolate oxidase (hydroxy acid oxidase 1, HAO1, EC 1.1.3.15) has been proposed as a target for substrate reduction therapy. Accumulation of the substrate of HAO1 is benign as the accumulated glycolate is highly soluble and can be excreted freely. Similarly, there is no damaging deficit of metabolites downstream of HAO1 as sufficient levels of glycine and pyruvate can be obtained through other pathways. The safety of HAO1 inhibition is further supported by reports of asymptomatic loss-of-function *HAO1* mutations in humans (Frishberg et al., 2014; Narasimhan et al., 2016; McGregor et al., 2020). Proof-of-concept rescue by HAO1 inhibition was shown in mouse models of primary hyperoxaluria by genetic knockout (Martin-Higueras, Luis-Lima, and Salido 2016; Zabaleta et al., 2019), RNAi knockdown (Dutta et al., 2016; Li et al., 2016), and small-molecule inhibition (Martin-Higueras, Luis-Lima, and Salido 2016). RNAi targeting HAO1 has also been validated in a non-human primate model (Liebow et al., 2017) and recently approved for the treatment of PH1 patients (Frishberg et al., 2014; Frishberg et al., 2021; Scott and Keam 2021).

HAO1 is a flavin mononucleotide (FMN)-dependent enzyme that oxidizes α-hydroxy acids to the corresponding α-keto acids, with the concomitant reduction of molecular oxygen to H_2_O_2_. Human HAO1 (hHAO1) exhibits a broad substrate range from the two-carbon glycolate to the 16-carbon 2-hydroxypalmitate, with glycolate being the preferred substrate (Murray et al., 2008). The other human isozyme HAO2 (long-chain hydroxy acid oxidase, or LCHAO) oxidizes only long-chain aliphatic α-hydroxy acids (e.g., 2-hydroxypalmitate, 2-hydroxyoctanoate) *in vitro* (Jones, Morrell, and Gould 2000), but its physiological substrate is not known. The two isozymes share 50% sequence identity (Supplementary Figure S1). Two key features differentiate HAO1 from HAO2: the presence of tryptophan (Trp110 in hHAO1) coordinating the glycolate substrate and an inserted “gating loop” (hHAO1 aa169-212, sometimes referred to as loop 4 due to its position between beta-strand 4 and helix 4) predicted to shield the active site during catalysis (Supplementary Figures S1, S2). This gating loop is highly flexible, as demonstrated by the variable orientation and degree of disorder observed in crystal structures, and is poorly conserved (in both sequence and length) within the hydroxy acid oxidase family (Supplementary Figure S2) (Murray et al., 2008).

Several published inhibitors of hHAO1, largely based on a heterocyclic carboxylic acid chemotype, have been reported from structure-guided design (Stenberg and Lindqvist 1997; Jones, Morrell, and Gould 2000; Murray et al., 2008; Bourhis et al., 2009), *in silico* docking (Bourhis et al., 2009) and phenotypic screens (Wang et al., 2016), although so far none have proceeded to clinical studies. Given the limited diversity of these available starting points, we performed a crystallography-based fragment screen (Cox et al., 2016; Hoffer et al., 2018), to identify new scaffolds and, potentially, novel binding pockets for inhibitor development. We identified and optimized hits bound to a previously uncharacterized non-orthosteric pocket of hHAO1, resulting in hHAO1 inhibition at µM potency. These molecules are dissimilar in chemotype from previously published inhibitors and do not compete with the substrate glycolate.

## Materials and Methods

### Chemicals

All fragments were purchased from Enamine. Follow-up compounds were purchased either from Enamine (active site compounds) or MolPort (gating loop site compounds).

### Expression, and Purification of hHAO1

A hHAO1 construct, encoding residues Met1-Ser368, with an engineered N-terminal His6-tag subcloned into the pNIC28-Bsa4 vector (Mackinnon et al., 2018), was transformed into *E. coli* BL21 (DE3) cells. hHAO1 was cultured in auto-induction Terrific Broth (Fox and Blommel, 2009) for 6 h before incubation at 18°C for 40 h. Cell pellets were harvested, homogenized in lysis buffer (50 mM HEPES pH 7.5, 500 mM NaCl, 5% glycerol, 0.5 mM TCEP, 0.1 mM FMN), and centrifuged to remove insoluble material. The supernatant was purified by Nickel affinity (Thermo Fisher Scientific) followed by size exclusion (Superdex 200 Hi-Load 16/60, GE Healthcare) chromatography into crystallization buffer (50 mM HEPES pH 7.5, 500 mM NaCl, 5% glycerol, 0.5 mM TCEP). The purified protein was concentrated to 13.7 mg/ml by cycles of centrifugation (15 min, 4,000 rpm, 4°C) and mixing in a Vivaspin protein concentrator with a molecular weight cut-off of 30 kDa (GE Healthcare).

### Crystallography-Based Fragment Screening

To launch the fragment soaking campaign, hundreds of crystals were grown by vapor diffusion at 4°C in 150 nL sitting drops of 13.7 mg/ml protein equilibrated against the well solution containing 25–35% PEG1000, sodium malonate-imidazole-boric acid (MIB) buffer, pH 8.0 (Mackinnon et al., 2018). For soaking, 50 nl of each fragment compound (∼500 fragments from the DSi-Poised Library (Cox et al., 2016); from supersaturated stock solutions of 100–500 mM in d6-DMSO, resulting in a final concentration of 25–125 mM fragment) was added to a crystallization drop using an ECHO acoustic liquid handler dispenser at the Diamond Light Source beamline I04-1 XChem facility. Crystals were soaked for 2 hours with fragments before being harvested using the SHIFTER technology, cryo-cooled in liquid nitrogen, and measured using the “automated unattended” mode of the I04-1 beamline. The XChemExplorer pipeline (Krojer et al., 2017) was used for structure solution with parallel molecular replacement using DIMPLE (Wojdyr et al., 2013), followed by map averaging and statistical modeling to identify weak electron densities generated from low occupancy fragments using PanDDA (Pearce et al., 2017). Model building and refinement were performed using the WinCoot and REFMAC software integrated into the XChemExplorer pipeline (Krojer et al., 2017). Figures were prepared using ICM-Pro software (Molsoft LLC). Coordinates and structure factors for all data sets with bound fragments are deposited in the RCSB Protein Data Bank. PanDDA electron density maps for HAO1 co-structures with fragments 1 - 6 are provided as Data Sheets 2 - 7 in Supplementary Material.

### Amplex Red Activity Assay

hHAO1 activity *in vitro* was determined using the Amplex Red fluorescence hydrogen peroxide assay (Sigma Aldrich). The Amplex Red assay reagent contained horseradish peroxidase (EC 1.11.1.7, 0.2 U/ml) and its substrate Amplex Red (10-acetyl-3,7-dihydroxyphenoxazine, 100 µM) in assay buffer. The assay buffer contained 50 mM sodium phosphate, pH 7.4, 200 mM KCl, 2 mM MgCl_2_, and 0.01% TritonX. Determination of suitable assay parameters is described in the Supplementary Material. To measure activity and inhibition of hHAO1, 10 µl/well of reaction containing 30 nM of hHAO1 and 30 µM glycolate in assay buffer was incubated with varying concentrations of fragment (1 and 10 mM) or follow-up compounds (0–1 mM, 12 concentrations), and dispensed into 384-well assay plates (Greiner^®^). Following 10 min incubation at room temperature, 10 µl/well of Amplex Red reagent was added. Fluorescence emission was measured at 585 nm, with excitation at 570 nm, after a further incubation period of 10 min, using a PHERAstar plate reader with a FI 540 590 optics module. All reactions were performed in technical triplicates for two different preparations of hHAO1. Reaction rate, defined as total H_2_O_2_ produced by the hHAO1 reaction, was determined from the H_2_O_2_ standard curve. Data were plotted using GraphPad Prism software; curve fitting was performed with nonlinear least-squares regression fit to log (inhibitor) vs response (three parameters) equation (for IC50 determination) and to mixed model, competitive, non-competitive, and uncompetitive inhibition models from the GraphPad Prism Enzyme kinetics–Inhibition equations and the best fit was selected for each ligand by comparison of Akaike’s information criterion probability scores and extra sum-of-squares F test *p* values calculated by the software (for inhibition mode determination). An additional parameter calculated when fitting to enzyme kinetics–mixed model inhibition equation was the α value. This value is defined as the difference between the inhibition constant for the free enzyme (K_i_) and the inhibition constant for the enzyme-substrate complex (αKi) and can be related to the initial velocity (V_0_), substrate concentration ([S]), inhibitor concentration ([I]), and other kinetic parameters (K_m_, V_max_, K_i_) as follows: V_0_ = V_max_ [S]/(K_m_ (1 + [I]/K_i_) + [S](1 + [I]/(*α*∗K_i_))). When an inhibitor binds with equal affinity to both the enzyme alone and the enzyme-substrate complex, *α* = 1, indicating a non-competitive inhibition mode (K_i_ = αK_i_). When an inhibitor preferentially binds to the enzyme alone, *α* > 1, with a very large value indicating an inhibition mode close to competitive inhibition (αK_i_ cannot be calculated for competitive inhibition as no binding to the enzyme-substrate complex occurs). When inhibitor preferentially binds to the enzyme-substrate complex, *α* < 1, with a very small value indicating an inhibition mode close to uncompetitive inhibition (K_i_ for the free enzyme cannot be calculated for uncompetitive inhibition as no binding to the free enzyme occurs).

### Surface Plasmon Resonance

Purified hHAO1 (30 μg/ml) was attached via the C-terminal His6-tag to a Ni-NTA chip to a density of 5000 RU. The assay buffer was 20 mM HEPES, pH 7.5, 0.05% TWEEN20, 200 mM NaCl, 0.5 mM TCEP, 5% DMSO. A serial dilution (11 concentrations) was prepared in the above buffer for each analyte (small molecule) by 1:1 dilution from 100 to 0.05 µM and the subsequent solutions were passed over the chip at a flow rate of 30 μL/min. Data are from an n = 1 experiment.

## Results

### Three Fragments Identified by X-Ray Crystallography to Bind HAO1

**FIGURE 1:**
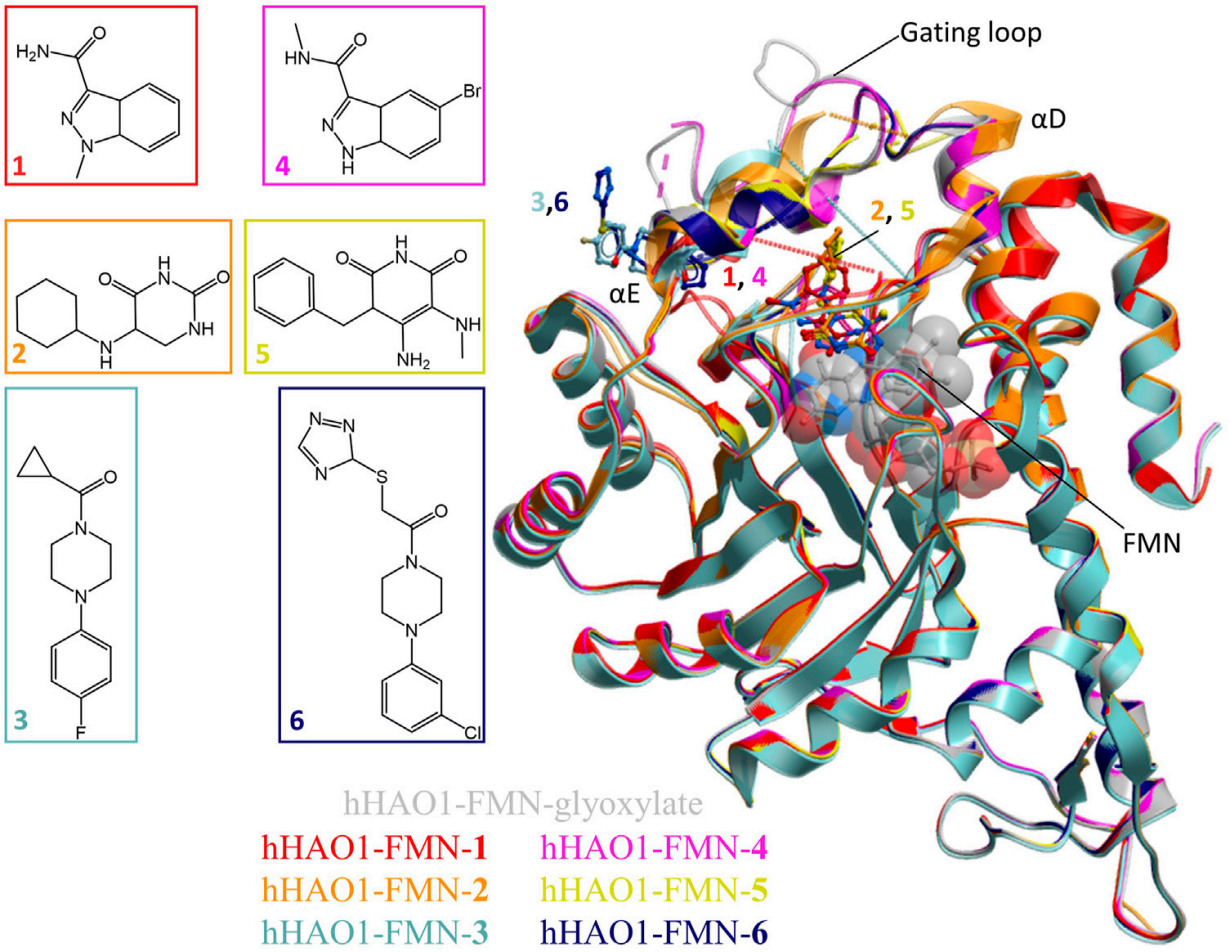
Fragment screening by X-ray crystallography. Superimposition of fragment-bound HAO1 structures. All structures contained FMN, shown as spacefill and sticks. Protein structures are displayed as ribbons and colored in the same scheme as their bound fragments. Dotted lines in structures indicate disordered protein regions. Fragments are displayed as sticks, colored according to the same key. Inset*:* Chemical structures of fragments bound to HAO1.

We applied crystallography-based fragment screening to determine hHAO1 crystal structures with novel ligands bound (Bradley et al., 2017). Among the different constructs tested, hHAO1 Met1-Ser368 (last 2 amino acids truncated) readily yields reproducible crystals with consistent diffraction quality better than 2 Å. Pre-formed hHAO1 crystals were each soaked with an individual fragment from the DSi-Poised fragment library (Cox et al., 2016) and subjected to high throughput X-ray crystallography to identify bound fragments (Supplementary Table S1) (Krojer et al., 2017; Pearce et al., 2017). Over 400 structures were determined by automated molecular replacement and refinement (Krojer et al., 2017) to 1.2–2.2 Å resolution. Examination of SigmaA-weighted (2mF_o_-F_c_) electron density maps, with background correction, performed using multi-crystal isomorphous difference density maps (PanDDA method; Pearce et al., 2017) reveal two fragments bound at the active site (**1, 2**) and one fragment at the surface-exposed gating loop, ∼12 Å from the active site (**3**) (Figure 1; ligand densities in 2F_o_-F_c_ maps before background (ground state) subtraction and 2mF_o_-F_c_ maps output from PanDDA are shown in Supplementary Figure S3). Each original chemotype was expanded by soaking with a structurally related fragment, yielding structures bound with a further two active site fragments (**4, 5**) and one gating loop fragment (**6**).

### Active Site Fragments Replicate Known Inhibitor Binding Mode

**FIGURE 2:**
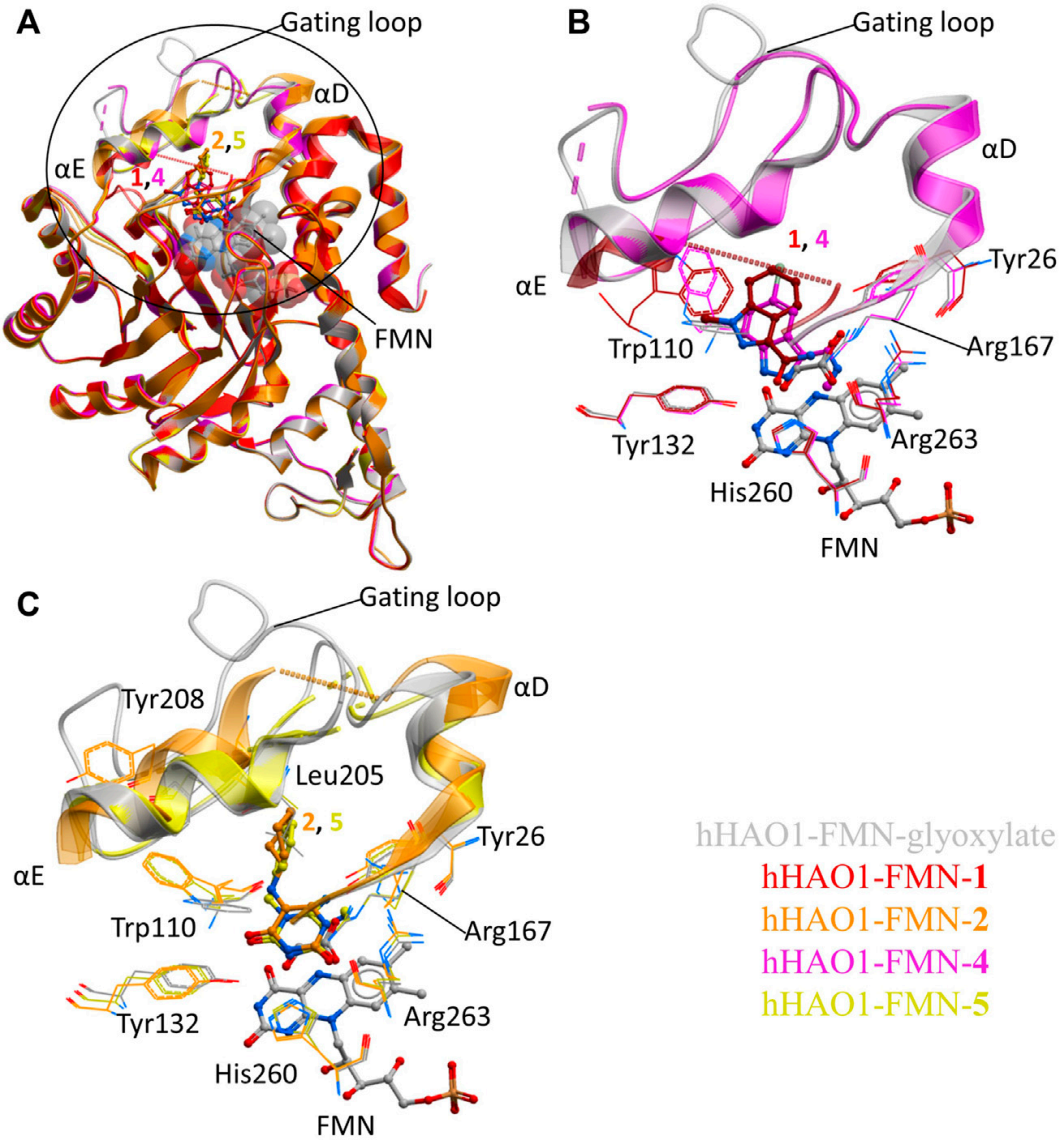
Fragment hits binding to the active site of hHAO1. **(A)** Superimposed structures of fragment-bound HAO1 structures. All structures contained FMN, one copy of which is shown as spacefill. Structures with fragments **1**, **2**, **4**, and **5** are colored red, orange, pink, and yellow respectively. Fragments are displayed as sticks, colored according to the same key. **(B)** Close-up view, showing key structural features and residues involved in binding fragments **1** (red) and **4** (pink) relative to glyoxylate-bound structure (gray). **(C)** Close-up view, showing key structural features and residues involved in binding fragments **2** (orange) and **5** (yellow) relative to glyoxylate-bound structure (gray).

The active site fragments **1**, **2**, **4**, and **5** occupy the glycolate binding site, stacking with the FMN co-factor (Figure 2). Fragments **1** and **4** have an indazole-carboxamide scaffold while fragments **2** and **5** have a 5-aryl-pyrimidine-2,4-dione scaffold. The overall fragment-bound structures are similar except for the region of the gating loop (Figure 2A). The variable conformation of the gating loop, as mentioned in the Introduction, is a characteristic feature of hHAO1 structures, observed across ligand-bound states, and reflects the flexibility of this region.

To contextualize the active site fragments, we first analyzed the binding modes of known HAO1 inhibitors, by comparing a 1.2 Å resolution structure of hHAO1 bound with 5-[(4-methylphenyl)sulfanyl]-1,2,3-thiadiazole-4-carboxylic acid (CCPST) determined in this study (PDB code 6gmc; Supplementary Table S2 and Figure S4), with reported structures of triazole, dioxo-pyrroline, benzoic acid, and indazole carboxylic acid inhibitors bound to either spinach glycolate oxidase (sGOX; PDB codes 1al7 and 1al8; Stenberg and Lindqvist, 1997) or hHAO1 (Supplementary Figure S5; PDB codes 2rdt (Murray et al., 2008), 2w0u (Bourhis et al., 2009), 6w44, 6w45, 6w4c (Lee et al., 2021) and 7m2o (Ding et al., 2021)), sGOX-FMN-glyoxylate (PDB code 1gox; Lindqvist, 1989) and hHAO1-FMN-glycolate (PDB codes 2nzl (Mackinnon et al., 2018) and 6gmb, determined in this study). These inhibitor-bound structures all demonstrate similar features: displacement of residues lining the substrate-binding pocket (Tyr26, Trp110, Tyr132, Arg167, Arg263) relative to glycolate/glyoxylate-bound hHAO1 structures, interaction with residues involved in substrate turnover (Asp160, Lys236, His260), and disruption of the hydrogen bonding network posited to maintain gating loop conformation during catalysis (Trp110, Tyr134, Leu191, Tyr208; (Murray et al., 2008)) (Supplementary Table S3 and Figure S5).

The active site fragments described in this work maintain similar binding modes to the reported inhibitors with respect to the heterocyclic polar head group that interacts with residues lining the substrate-binding pocket and causes rotation of the Trp110 sidechain outward from the active site, and the attached non-polar group that disrupts the hydrogen bonding network involved in maintaining gating loop conformation for catalysis (Figure 2).

Fragment **1** is less embedded in the active site than the described indazole carboxylic acid inhibitors (Supplementary Figure S5F), likely binding less tightly but still causing significant displacement of Trp110 and interacting with His260 and Tyr132 via the carboxamide group (Figure 2B). The related, larger fragment, **4**, superimposes well with published indazole and triazole carboxylic acid inhibitors, such as 4-carboxy-5-dodecylsulfanyl-1,2,3-triazole (Supplementary Figure S5C,F), with the methyl-carboxamide group in place of the carboxylic acid. The methyl-carboxamide group of **4** interacts with the substrate-binding residues Tyr26, Arg167, and Arg263; the pyrazole nitrogen atoms hydrogen bond with His260 and Tyr132; and the bromo-phenyl group displaces Tyr110, causing it to rotate 180° out from the active site (Figure 2B).

Fragments **2** and **5** recapitulate the binding pose seen in the published structure of sGOX bound to a dioxo-pyrroline inhibitor (3-decyl-2,5-dioxo-4-hydroxy-3-pyrroline, TKP, PDB code 1al7, Supplementary Figure S5E (Stenberg and Lindqvist 1997)), coordinated by Tyr132, Arg167, His260 and Arg263 in the substrate-binding pocket (Figure 2C; Supplementary Figure S5D). Binding of **2** and **5** also causes the Trp110 sidechain to rotate 180° out from the active site as seen in other hHAO1-inhibitor complexes (Figure 2C). In both fragments, the hydrophobic group attached to the piperidine (cyclohexane in **2** and phenyl in **5**) makes few interactions but in fragment **2** it displaces Tyr208, further disrupting the hydrogen bonding network around the gating loop.

Collectively these four fragments represent embellishment to known inhibitor scaffolds that have not been explored, and we next aimed to characterize their utility as starting points for inhibitor development by characterizing them in solution.

### Novel Non-orthosteric Binding Pocket at the Gating Loop

**FIGURE 3:**
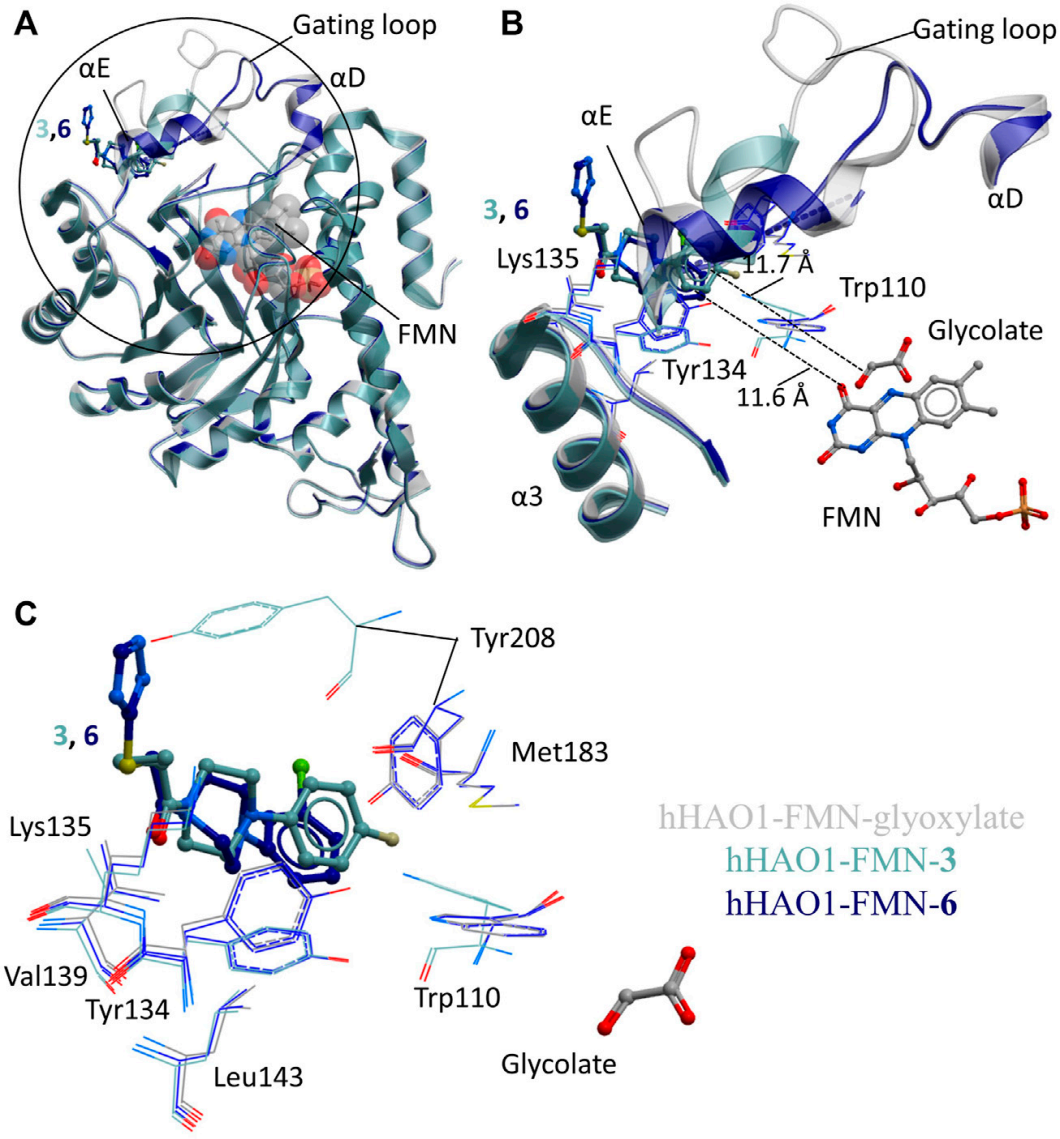
Fragment hits binding to the gating loop site of hHAO1. **(A)** Superimposition of fragment-bound hHAO1 structures. FMN is shown as spacefill and sticks. Structures with fragments **3** and **6** are colored light and dark blue respectively. Fragments are displayed as sticks, colored according to the same key. **(B-C)** Close-up view of HAO1 gating loop site, circled in panel A, showing key structural features and residues involved in binding fragments **3** (light blue) and **6** (dark blue) relative to glyoxylate-bound structure (gray). (B) shows secondary structures forming the gating loop pocket as ribbons, key interacting residues as lines, and fragments, and FMN as sticks. The shortest distance from fragments to glyoxylate is indicated by dotted lines. **(C)** shows interacting residues that interact with fragments **3** and **6** as lines and glycolate and fragments are shown as sticks.

The two non-orthosteric fragments **3** and **6**, bound above the active site where the gating loop would otherwise be, contain a phenylpiperazine scaffold (Figure 3A). The phenylpiperazine moiety fits into a hydrophobic groove formed by the regions containing loop 3/helix α3 (aa131-150; Tyr134, Val139, Leu143) and gating loop (loop 4)/helix αE (aa171-213; Met183, Tyr208) (Figure 3B).

Binding of **3** causes small movements to the sidechain of Tyr134 in loop 3 to avoid clashing with the fluorophenyl group of the fragment. This disrupts the hydrogen bonding network between Tyr134, Trp110, Leu191, and Tyr208, causing additional minor displacement of the Trp110 sidechain (Figure 3C). Loss of these hydrogen bonds, combined with the position of the piperidine core in the space usually occupied by the gating loop, causes helix αE to shift so that Tyr208 is now mostly surface exposed and interacting with the fragment. The carbonyl group of **3** also hydrogen bonds with the backbone nitrogen of Lys135.

Binding of **6** also causes movement of the Tyr134 sidechain (both conformations are observed) and maintains the hydrogen bond with the backbone nitrogen of Lys135 but the sulfanyl-triazole extension prevents movement of Tyr208 to the location as seen with **3** and instead the piperazine ring is twisted slightly to avoid clashing (Figure 3C).

We next asked whether this gating loop pocket had been observed indirectly in structures of HAO1 (spinach or human) bound to active site inhibitors. We inspected structures of HAO1 bound to active site inhibitors that contained a secondary moiety, away from the heteroaryl-carboxylic acid, that could theoretically reach the gating loop pocket described in this work. Of the seven compounds that could occupy the gating loop pocket (Supplementary Figure S6), only one does so. This compound was reported as a dual lactate dehydrogenase (LDH)-HAO1 inhibitor (5-[(5'-{1-(4-carboxy-1,3-thiazol-2-yl)-5-(cyclopropylmethyl)-4-[(3-fluoro-4-sulfamoylphenyl)methyl]-1H-pyrazol-3-yl}-2′-fluoro [1,1′-biphenyl]-4-yl)oxy]-1H-1,2,3-triazole-4-carboxylic acid; PDB code 7m2o (Ding et al., 2021)), and the group occupying this pocket is the thiazole-carboxylic acid component of the LDH-targeting moiety (Supplementary Figure S6C).

### Fragment Characterization in Solution

**FIGURE 4:**
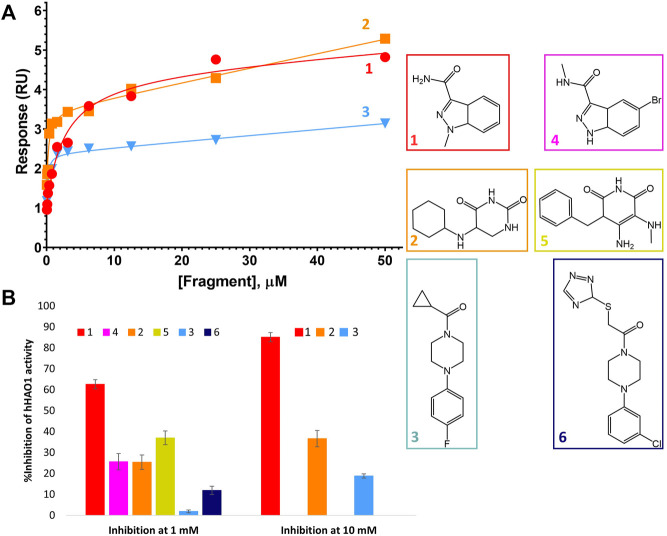
Binding to and inhibition of hHAO1 by fragments. **(A)** Characterization of fragment binding to hHAO1 by surface plasmon resonance. Plot of relative response, in response units (RU), against fragment concentration. Response curves for fragments **1-3** are shown in red, orange, and blue respectively. **(B)** Bar chart showing the observed change in hHAO1 activity in the presence of 1 mM or 10 mM fragment, as measured in the Amplex Red activity assay. Change in activity is reported as %Inhibition. Errors bars are standard deviation of three replicates. *Inset:* Fragment chemical structures.

After identification of six fragment hits *in crystallo*, we next characterized their binding and inhibition of hHAO1 in solution. We first determined the binding affinity of the control inhibitor CCPST for hHAO1, which has not previously been reported for any HAO enzyme, using surface plasmon resonance (SPR). We immobilized His-tagged hHAO1 to an Ni-NTA coated SPR chip and passed increasing concentrations (0–100 μM, 11 concentrations) of CCPST across it at a flow rate of 30 μl/min. Using this method, we measured a K_D_ of 47.5 µM for CCPST (Supplementary Figure S7), in line with the observed potency, validating our set-up for measuring the affinity of novel fragments and compounds. Here we find that the three original fragments (**1**–**3**) demonstrated measurable, specific binding to hHAO1 in solution at concentrations from 15 to 50 μM, though the binding was too weak to determine K_D_ values (Figure 4A).

We next measured enzyme activity of our hHAO1 preparation, as well as inhibition by CCPST and potentially our fragment hits, using the previously established horseradish peroxidase-coupled assay employing an Amplex Red reporter system (Wang et al., 2016, Supplementary Figure S8A). After establishing the incubation period and linear assay window for the reaction (Supplementary Figure S8B,C), hHAO1 activity was confirmed by titrations with the favored substrate glycolate (calculated K_m_ of 18.5 µM; Supplementary Figure S8D) and the alternate substrate 2-hydroxypalmitate (100-fold higher K_m_; Supplementary Figure S8E).

HAO inhibition by CCPST has previously been reported for purified mGO (IC_50_ 43–198 µM) (Martin-Higueras, Luis-Lima, and Salido 2016; Moya-Garzón et al., 2018), rat LCHAO (equivalent to hHAO2) (IC_50_ 3.6 µM), flavin dehydrogenase domain of yeast flavocytochrome b2 (IC_50_ 6 µM), and hHAO1 (IC_50_ 4.5 µM) (Chen et al., 2012). However, puzzlingly, published kinetics of CCPST inhibition against these targets report a non-competitive inhibition mode (Chen et al., 2012; Martin-Higueras, Luis-Lima, and Salido 2016), which would indicate non-orthosteric binding, contradicting the active site binding observed in the three published HAO-CCPST structures (PDB codes 3sgz, 2w0u, and 6gmc). Inhibition of hHAO1 by CCPST observed in this work is consistent with these published values, with an average IC_50_ value of 22 ± 9 µM across three preparations of hHAO1 (Supplementary Figure S9A). In this work, we definitively observed a competitive inhibition mode (Supplementary Figure S9B), consistent with the orthosteric binding expected from the crystal structures.

We then measured inhibition of hHAO1 activity by the fragments (Figure 4B), showing that the active site fragment **1** had an IC_50_ of 420 µM whereas the remaining active site fragments, **2**, **4**, and **5**, demonstrated 10, 26, and 37% inhibition respectively at 1 mM. The loop site fragments **3** and **6** showed 2% and 12% inhibition respectively at 1 mM, with fragment **3** showing 19% inhibition at 10 mM.

The above-observed binding and inhibitor effects prompted us to perform one round of optimization to improve fragment potency. Of the different approaches described to optimize fragment hits (Joseph-McCarthy et al., 2014), we adopted the approach of fragment growing, in the absence of groups of nearby fragments suited to the alternative fragment linking or merging approaches.

### Optimizing Active Site Fragments Into µM Potency Inhibitors

**FIGURE 5:**
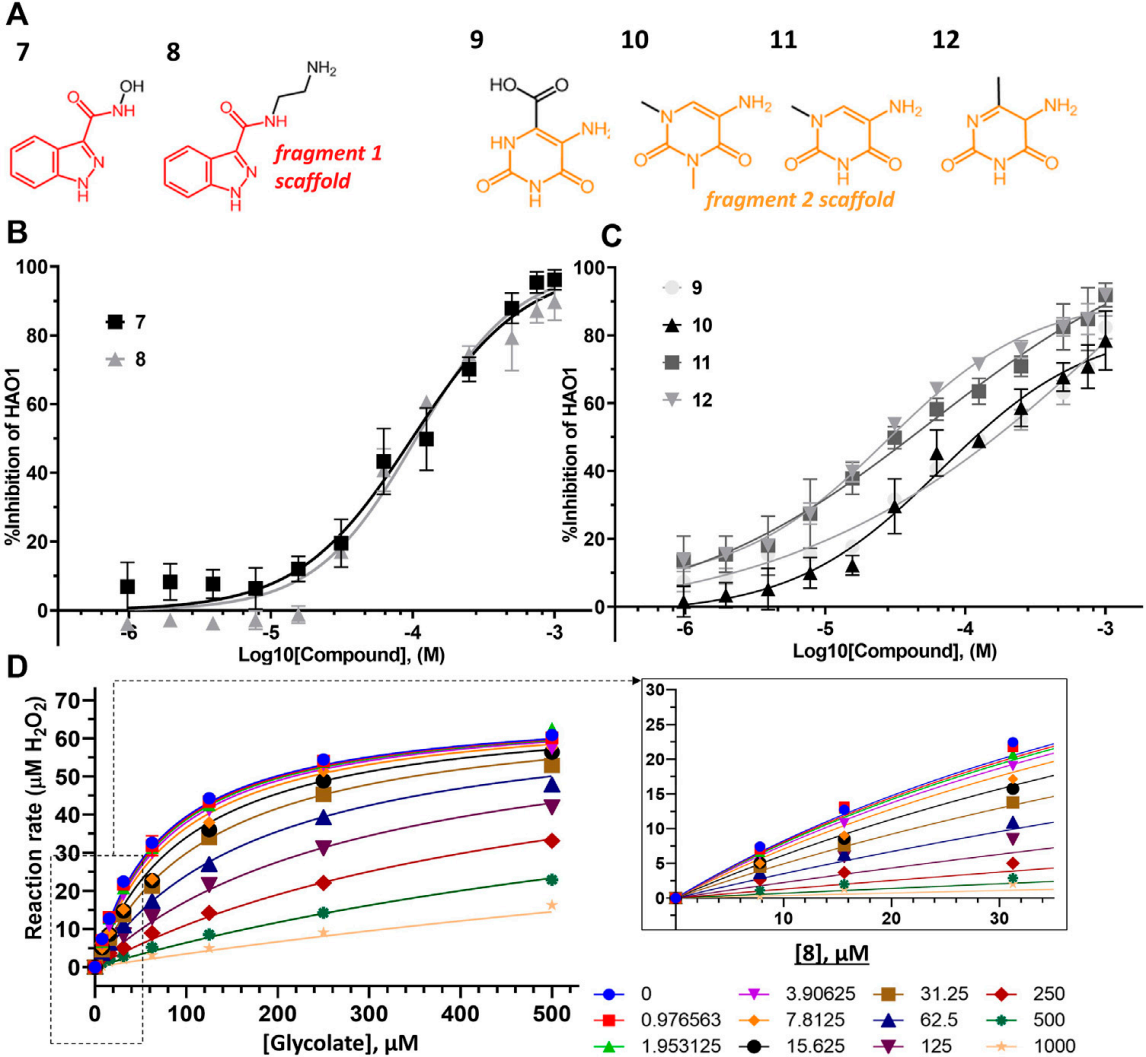
Binding to and inhibition of hHAO1 by follow-up compounds from the active site. **(A)** Chemical structures of follow-up compounds derived from active site fragments. Fragment **1** scaffold is colored red, and fragment **2** scaffold is colored orange. **(B)** Concentration-response curve for inhibition of hHAO1 by compounds **7** and **8** measured in the Amplex Red activity assay at 30 µM glycolate. Error bars are standard deviation of three replicates. **(C)** Concentration-response curve for inhibition of hHAO1 by compounds **9–12** measured in the Amplex Red activity assay at 30 µM glycolate. Error bars are standard deviation of three replicates. **(D)** Least-squares nonlinear fit of hHAO1 reaction rate (total H_2_O_2_ produced after 15 min reaction, µM) against increasing glycolate concentrations (0–500 µM) in the presence of different concentrations of compound **8** (0–1 mM). Curves were fitted to the competitive inhibition model, the best fitting Enzyme kinetics–Inhibition equation, in GraphPad Prism. Inset: Close-up view of plot showing hHAO1 reaction rate (total H_2_O_2_ produced after 15 min reaction, µM) against increasing glycolate concentrations (0–35 µM) in the presence of different concentrations of compound **8** (0–1 mM).

For fragments binding at the active site, we purchased 40 follow-up compounds from the Enamine Building Blocks commercial library, aiming to explore subtle changes to the two fragment scaffolds. We focused on ring additions at different positions of the fragment **1** scaffold and substitution of the cyclohexane (that showed no interaction with the protein) for the fragment **2** scaffold. Compounds were screened for inhibition of hHAO1 in the Amplex red assay at 1 mM concentration. Half-maximal inhibitory concentration (IC_50_) and (Astex therapeutics lipophilic ligand efficiency score (LLE_AT_; (Mortenson and Murray 2011)) values were determined for compounds showing good inhibition (chemical structures shown in Figure 5A).

Removal of the methyl group from the pyrazole and addition of a hydroxyl (compound **7**; IC_50_ 153 μM; LLE_AT_ 0.40 kcal/mol) or an amino-ethyl (compound **8**; IC_50_ 81 μM; LLE_AT_ 0.47 kcal/mol) to the carboxamide of fragment **1** provided a 5-fold increase in potency (Figure 5B; Table 1). Surprisingly, a huge increase in potency was observed relative to the fragment **2** scaffold by removing the cyclohexane and substituting the piperidine with methyl groups, yielding four compounds with IC_50_ < 100 µM and LLE_AT_ > 0.7 kcal/mol. Specifically, substitution of the piperidine with 6-carboxylate (compound **9**; IC_50_ 93 μM; LLE_AT_ 0.71 kcal/mol), 1,3-dimethyl (compound **10**; IC_50_ 60 μM; LLE_AT_ 0.77 kcal/mol), 1-methyl (compound **11**; IC_50_ 40 μM; LLE_AT_ 0.88 kcal/mol), or 6-methyl (compound **12**; IC_50_ 27 μM; LLE_AT_ 0.89 kcal/mol) substituents result in a more than 10-fold increase in potency (Figure 5C; Table 1).

**TABLE 1:** Kinetic parameters for inhibition of hHAO1 by follow-up compounds 7–14.95% confidence intervals (CI) and best-fit values for IC_50_, K_i,_ and α were determined by fitting log [inhibitor] vs. response (%inhibition) curves. Best-fit values for IC_50_ are those reported in the text. Best-fit values for K_m_ and V_max_ were determined by individual fitting of Michaelis-Menten curves—[glycolate] vs. response (reaction rate, glycolate consumed)—in the presence and absence of 1 mM compound.

	95% CI IC_50_, µM (Best-fit)	95% CI K_i_, µM (Best-fit)	95% CI α (Best-fit)	K_m_, µM, Best-fit		V_max_, µM, Best-fit	
				None	1 mM	None	1 mM
Active site fragment **1** (indazole carboxamide)-derived compounds							
**7**	110.3–215.2 (152.9)	56.73–64.25 (60.37)	NA	64.62	811.2	62.16	47.76
**8**	57.1–117.1 (81.26)	37.72–44.32 (40.89)	NA	67.98	1,193	69.00	54.60
Active site fragment **2** (dioxo-pyrimidine)-derived compounds							
**9**	48.1–193.5 (93.35)	113.1–125.9 (119.4)	NA	63.65	572.3	57.40	56.25
**10**	39.5–92.0 (59.78)	157.4–177.2 (166.9)	NA	53.34	304.65	60.08	56.47
**11**	22.8–71.1 (39.77)	219.2–247.8 (233.0)	NA	48.89	270.1	55.07	55.85
**12**	19.9–38.0 (27.40)	141.4–155.9 (148.4)	NA	55.39	395.7	56.49	54.05
Gating loop fragment **3** (phenylpiperazine)-derived compounds							
**13**	27.4–71.4 (43.83)	59.07–82.92 (69.7)	7.84–27.32 (13.27)	61.73	279.1	67.02	27.28
**14**	100.5–255.7 (157.5)	83.06–118.7 (98.9)	9.41–59.28 (18.4)	70.70	349.7	73.49	43.12

Next, we measured an array of compound concentrations (0–1 mM) versus glycolate concentrations (0–500 µM) to determine the inhibition mode of these improved compounds. As expected, all compounds derived from active site fragments demonstrate competitive inhibition mode with respect to glycolate, entirely consistent with their binding in the substrate-binding pocket (exemplified by **8**, Figure 5D; further data in Supplementary Figure S10).

### Optimizing Gating Loop Site Fragments Into µM Potency Inhibitor

**FIGURE 6:**
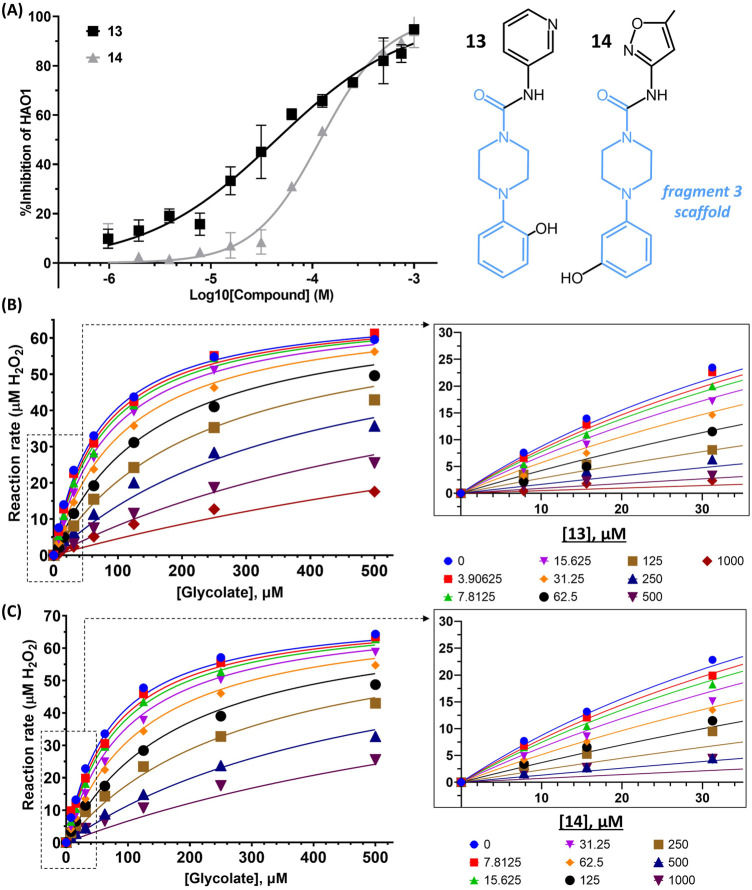
Binding to and inhibition of hHAO1 by follow-up compounds from the gating loop site. **(A)** Concentration-response curve for inhibition of hHAO1 by compounds **13** and **14** measured in the Amplex Red activity assay at 30 µM glycolate. Error bars are standard deviation of three replicates. *Inset*: Chemical structures of compounds **13** and compound **14**, fragment **3** scaffold is in blue. **(B–C)** Least-squares nonlinear fit of hHAO1 reaction rate (total H_2_O_2_ produced after 15 min reaction, µM) against increasing glycolate concentrations (0–500 µM) in the presence of different concentrations of compound **13** (0–1 mM; B) or compound **14** (0–1 mM; C). Curves were fitted to mixed inhibition model, the best fitting Enzyme kinetics–Inhibition equation, in GraphPad Prism. Inset: Close-up view of plot showing HAO1 reaction rate (total H_2_O_2_ produced after 15 min reaction, µM) against increasing glycolate concentrations (0–35 µM) in the presence of different concentrations of compound **13** (0–1 mM; B) or compound **14** (0–1 mM; C).

To generate analogs based on fragments at the gating loop site, we searched commercial catalogs through MolPort for compounds containing the scaffold of fragment **3** as a substructure, with the expectation that such compounds could improve affinity while maintaining binding at the gating loop pocket. Screening of these analogs using the Amplex Red activity assay, as described for the active site follow-up compounds, led to the identification of two promising hit compounds, **13** and **14**, with IC_50_ values of 44 μM and 158 µM and LLE_AT_ scores of 0.30 kcal/mol and 0.25 kcal/mol, respectively (Figure 6A).

We next investigated whether the inhibitory effect of compounds **13** and **14** was mediated by binding to the active site (akin to published inhibitors), or a non-orthosteric site (such as that revealed from the parent fragment’s co-structure). Glycolate-titration experiments with compound **13** showed a concentration-dependent decrease in V_max_, up to 2.8-fold reduction at 1 mM compound (*p* < 0.0001) and a concentration-dependent increase in K_m_, up to 4-fold increase at 1 mM compound (*p* < 0.0001), collectively indicating mixed model inhibition with respect to glycolate by compound **13**, which is supported by fitting in GraphPad Prism (Figure 6B; Table 1). Similarly, glycolate-titration experiments with compound **14** showed a concentration-dependent reduction in V_max_, up to 1.7-fold decrease at 1 mM compound (*p* < 0.0001) and a concentration-dependent increase in K_m_, up to 4.9-fold increase at 1 mM compound (*p* < 0.0001), supporting mixed model inhibition, as observed when fitting in GraphPad Prism (Figure 6C; Table 1). Fitting of glycolate-titration curves for both compounds yield alpha (*α*) values (indicating the difference between binding to the enzyme alone (K_i_) and binding to the enzyme-substrate complex (αK_i_), see materials and methods for details) of 13 and 18 for compounds **13** and **14,** respectively, which indicates both compounds bind more readily in the absence of substrate (Table 1).

Therefore, our competition data indicate that compounds **13** and **14** can bind to either hHAO1-FMN or hHAO1-FMN-glycolate complex, with a preference for the holoenzyme (*α* > 1), and in doing so reduce both substrate turnover (decreased V_max_) and hHAO1 affinity for glycolate (increased K_m_).

## Discussion

Inhibition of hHAO1 is a promising and recently validated approach for the treatment of primary hyperoxaluria. There is also potential to inhibit hHAO1 in the treatment of multifactorial kidney stone formation disorders as approximately 12% of the world population will suffer from kidney stone disease (urolithiasis) within their lifetime and 76% of these stones contain oxalate (Lowther, Holmes, and Yohannes 2017; Huang et al., 2020).

Currently, the only approved therapeutic modality for hHAO1 inhibition is RNAi. Existing small molecules in development include chemical series of pyrazole (Barawkar et al., 2012; Chen et al., 2012; Bourhis et al., 2009), triazole (Stenberg and Lindqvist 1997; Murray et al., 2008), or salicylate (Moya-Garzón et al., 2018) backbones and generally consist of a carboxylic acid like moiety mimicking the substrate carboxylic acid and one or more heterocyclic rings that pi stack with FMN (recently reviewed by (Moya-Garzon et al., 2021)).

Aside from the lack of diversity in these chemical series, their therapeutic use is limited by the metabolic instability, poor cell permeability, and cellular toxicity associated with carboxylic acid-containing drugs (Ballatore, Huryn, and Smith 2013), and consequently, no small-molecule inhibitor of hHAO1 has yet advanced to clinical trials in primary hyperoxaluria patients. Considering these limitations, we set out to identify new starting points for inhibitor development using fragment screening by X-ray crystallography. Our fragment screen identified two new active sites targeting scaffolds and a novel allosteric binding site with mid-micromolar affinity that hold promise for the development of selective and potent hHAO1 inhibitors.

Fragment **1** and its derivatives (**4**, **7**, **8**) are superficially similar to published pyrazole inhibitors in structure except that, instead of a carboxylic acid, they contain a carboxamide (**1**), methyl-carboxamide (**4**), hydroxy-carboxamide (**7**), or aminoethyl-carboxamide (**8**) group as part of the polar head group and the hydrophobic moiety is a phenyl ring fused to the pyrazole. Exploration of similar rLCHAO inhibitors showed no activity upon similar modification of the pyrazole ring and a 5-fold reduction in potency with a carboxamide (Barawkar et al., 2012), which may explain why carboxamide derivatives of hHAO1 inhibitors have not previously been explored despite the potential improvements in metabolic stability, cellular toxicity and permeability relative to the current carboxylic acid-containing compounds (Ballatore, Huryn, and Smith 2013). This work, however, demonstrates carboxamide derivatives can serve as highly efficient starting points for hHAO1 inhibitor development.

Fragment **2** and its derivatives (**5**, **9–12**) are somewhat similar to published diketone scaffolds (Rooney et al., 1983; Stenberg and Lindqvist 1997) but piperidines rather than pyrroles. Fragments **2** and **5** validate the diketone N-substituted ring scaffold for inhibition of hHAO1. These results suggest that the published dioxo-pyrroles described as inhibitors of the plant (Stenberg and Lindqvist 1997), pig (Rooney et al., 1983), and rat (Rooney et al., 1983) HAO enzymes may also inhibit hHAO1 and could be used as starting points for therapeutic inhibitor development. It is interesting to note that the most potent compound of this set (**12**) had a 6-methyl substituent and potency was 3-fold lower with a carboxylic acid, the traditional HAO1 binding functional group, at this position (**9**). Further structural work will be needed to unravel the interactions made by these very small fragments to guide their optimization.

While these new compounds provide a much-needed increase in scaffold diversity of hHAO1 inhibitors, their orthosteric binding mode means that they are unlikely to be selective against hHAO2, which could be important to reduce toxicity associated with cross-reactivity (Mattu et al., 2016). Screening methods that are independent of enzymatic activity and known binding sites, such as X-ray crystallography, are useful in identifying less conserved pockets, to reduce the risk of poor target specificity often associated with active site inhibitors. Discovery of fragments **3** and **6**, which bind at the highly variable gating loop, validates this approach for hHAO1. Sequence alignment shows poor conservation of residues at this site between hHAO1 and hHAO2 (Supplementary Figure S1).

This gating loop pocket does not overlay with the hydrophobic portions of the described HAO inhibitors, but a small part of a recently published dual HAO1-LDH inhibitor does reach this pocket. This supports merging of a gating loop “selectivity” moiety to an active site “potency” moiety to generate the next generation of HAO1 inhibitors and suggests chemistry to direct groups from the active site to the gating loop. For example, the cyclopropyl group of fragment **3** could be joined to the nitrogen at position 1 of the central pyrazole of the described HAO1-LDH inhibitor and the extraneous substitutions on the central pyrazole removed (Supplementary Figure S6).

Collectively the two fragment-bound structures (**3**, **6**), combined with the enzyme kinetics of follow-up compounds (**13**, **14**), indicate a persistent, true allosteric site at the gating loop that is a novel target for developing hHAO1 inhibitors to treat primary hyperoxaluria. A more specific and thorough investigation optimizing compounds **13** and **14** would likely yield potent, selective lead candidates for the development of therapeutic hHAO1 inhibitors for primary hyperoxaluria, and as such these early compounds present considerable utility for developing molecules distinct in both chemical scaffold and mechanism of action from all previously known inhibitors.

## Data Availability

The datasets presented in this study can be found in the Protein Data Bank (http://www.wwpdb.org). The accession numbers are 5qih, 5qib, 5qic, 7r4n, 7r4p, 7r4o, 6gmc.
