# Supplementary material for: Novel Starting Points for Human Glycolate Oxidase Inhibitors, Revealed by Crystallography-Based Fragment Screening
Source: Front Chem. 2022 May 4;10:844598. doi: 10.3389/fchem.2022.844598 (PMC9114433; doi:10.3389/fchem.2022.844598)
Supplement: Supplementary file 4 [file DataSheet1.PDF]

Supplementary Material

1 Sequence alignments of hydroxyacid oxidase family members

Figure S1: Sequence alignment of HAO1 and HAO2 orthologues. Sequence alignment of three short-chain 2-hydroxyacid oxidases (human, mouse and spinach HAO1) and three medium/long-chain 2- hydroxyacid oxidases (human, rat and fruit fly HAO2), prepared using the MultiAlin (Corpet 1988) and ENDscript (Robert and Gouet 2014) servers. Invariant residues are shown as white characters on a red background, residues that are similar in >70% of sequences are shown as bold characters on a yellow background and residues that are similar in >50% of sequences also have a yellow background. The N-terminal extension is boxed in blue and the gating loop in purple. A red asterisk marks hHAO1 Trp110.

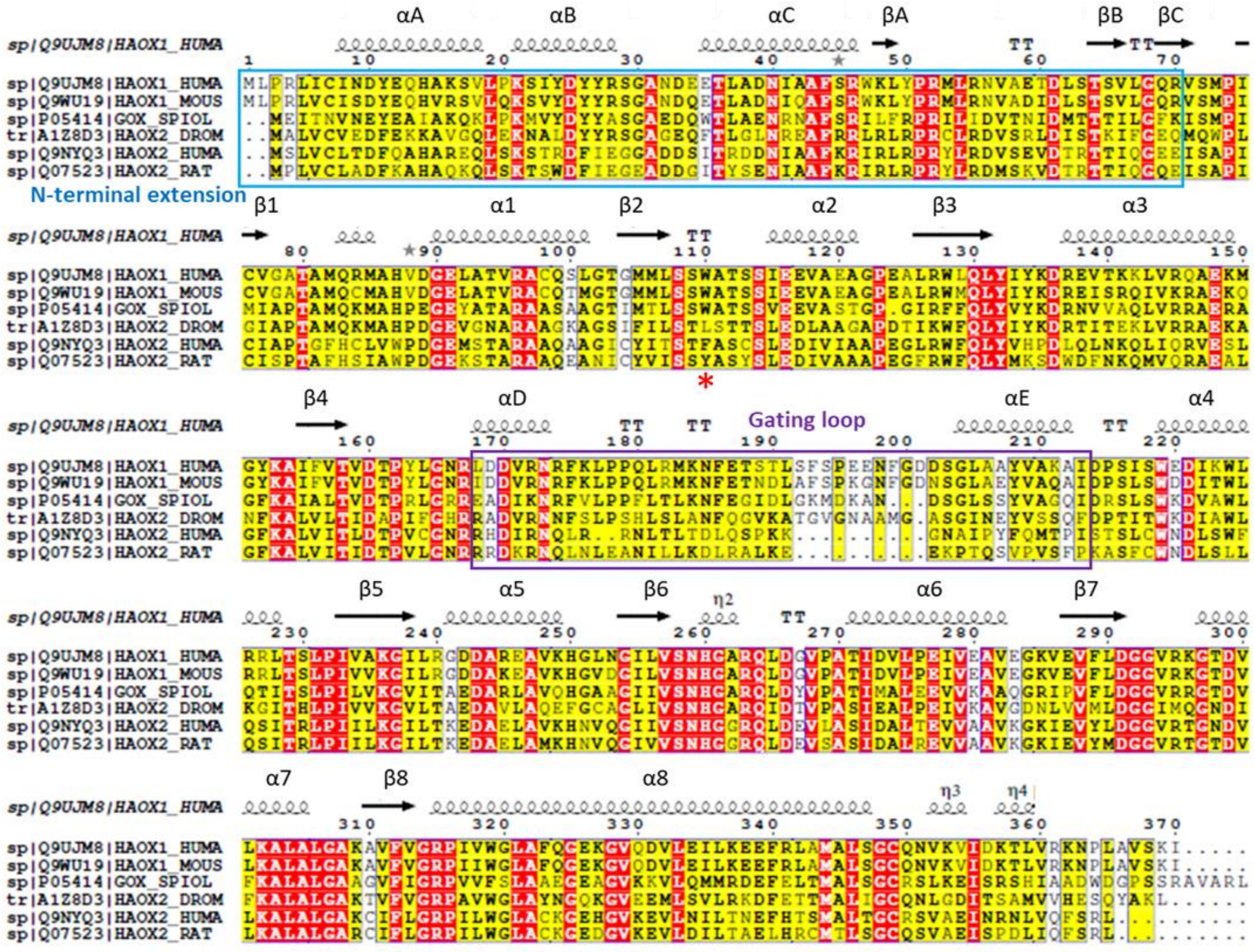

Figure S2: Sequence alignment of representative members of the (S)-2-hydroxyacid oxidase family. Sequence alignment of human hHAO1, human HAO2, lactate oxidase domain of yeast CYB2 and mandelate dehydrogenase (MDHC1) of A. thaliana, prepared using the MultiAlin (Corpet 1988) and ENDscript (Robert and Gouet 2014) servers. Invariant residues are shown as white characters on a red background, residues that are similar in >70% of sequences are shown as bold characters on a yellow background and residues that are similar in >50% of sequences also have a yellow background. The N-terminal extension is boxed in blue and the gating loop in purple. A red asterisk marks hHAO1 Trp110.

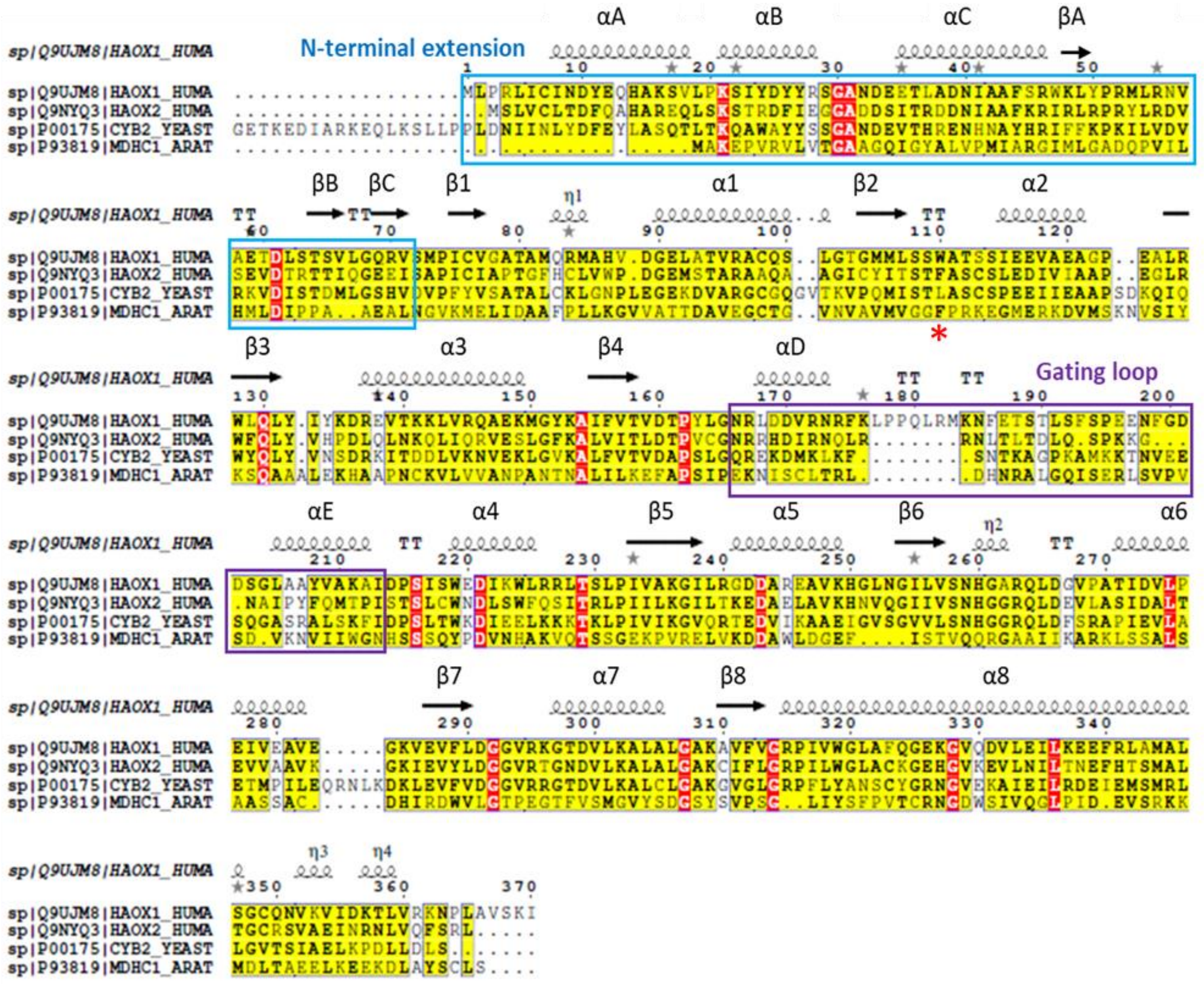

2 Refinement statistics for hHAO1 co-crystal structures solved in this work

Table S1: Crystallographic refinement statistics for hHAO1 co-crystal structures. Data for the highest resolution shell are shown in parentheses. All structures contained FMN. All structures were collected at the i04-1 beamline with a wavelength of 0.91587 and were in space group I4 with unit cell angles α= β = γ = 90°. *Four values – minimum, median, 95th percentile and maximum – are given for B factors.

Ligand:	Fragment 1	Fragment 2	Fragment 3	Fragment 4	Fragment 5	Fragment 6
**Unit cell: a, b, c (Å)**	97.46 97.46 80.52	97.59 97.59 80.87	97.00 97.00 80.64	97.13 97.13 80.84	97.06 97.06 80.22	97.06 97.06 80.22
**Resolution (Å)**	38.33 – 1.33	26.43 – 1.48	48.79 – 1.34	62.01 – 1.46	62.13 – 1.37	40.11 – 1.29
**Observed/ Unique reflections**	437306/ 84166 (14118/7122)	386723/ 63919 (26506/6325)	440078/ 81913 (15596/6324)	381858/ 64628 (25914/6439)	424651/ 77167 (19553/6662)	442261/ 88616 (9902/ 6345)
**R-merge**	0.04526 (0.6312)	0.03872 (0.3422)	0.0472 (0.6642)	0.05746 (0.9356)	0.03251 (0.7318)	0.04631 (0.4941)
**CC (1/2)**	0.999 (0.552)	0.999 (0.929)	0.999 (0.623)	0.999 (0.601)	1 (0.549)	0.999 (0.596)
**I/sig(I)**	17.09 (1.18)	23.46 (3.40)	18.12 (1.28)	14.53 (1.19)	20.29 (1.14)	15.60 (1.04)
**Completeness**	97.65 (82.88)	99.45 (99.14)	96.50 (74.94)	99.85 (99.77)	98.11 (84.84)	94.97 (68.19)
**Multiplicity**	5.2 (2.0)	6.1 (4.2)	5.4 (2.5)	5.9 (4.0)	5.5 (2.9)	5.0 (1.6)
**Rwork (%)**	18.6	19.0	19.3	21.3	14.6	14.5
**Rfree (%)**	19.9	20.7	20.7	23.6	17.1	16.5
**Rmsd bond length (Å)**	0.017	0.014	0.017	0.019	0.014	0.015
**Rmsd bond angle (°)**	1.86	1.58	1.78	1.44	1.51	1.54
**Clashscore**	2.95	1.84	2.55	6.78	5.51	6.42
**B factors (protein*, Å^2)**	**12, 18, 27, 40**	**12, 18, 29, 44**	**12, 20, 38, 50**	**12, 20, 38, 50**	**13, 22, 40, 56**	**9, 16, 31, 47**
**B factor (FMN*, Å^2)**	**12,16,20,22**	**17,19,21,21**	**10,13,17,18**	**13,19,22,23**	**14,19,23,24**	**10,16,19,22**
**B factor (fragment*, Å^2)**	**20,26,29,29**	**12,18,26,26**	**23,26,29,29**	**22,29,38,44**	**26,34,48,48**	**23,40,49,55**
**PDB Code**	**5qih**	**5qib**	**5qic**	**7r4n**	**7r4p**	**7r4o**

Table S2: Crystallographic refinement statistics for hHAO1-FMN-CCPST. Data for the highest resolution shell are shown in parentheses. Structure was collected at the Diamond Light Source synchrotron beamline i03 at a wavelength of 0.97625. PDB code: 6gmc. *Four B values – minimum, median, 95th percentile and maximum – are given for B factors.

Unit cell dimensions	(Å)	97.43 97.43 80.44
	(°)	90 90 90
Space group		I4
Resolution (Å)		31.02 - 1.2
Observed/Unique reflections		476163/115886 (45385/11609)
R-merge		0.0777 (0.6564)
CC (1/2)		0.993 (0.705)
I/sig(I)		8.96 (1.90)
Completeness		99.47 (99.28)
Multiplicity		4.1 (3.9)
Rwork (%)		13.4
Rfree (%)		15.5
Rmsd bond length (Å)		0.009
Rmsd bond angle (°)		1.37
Clashscore		10.48
**B factors (protein*, Å^2)**		8, 15, 32, 47
**B factor (FMN*, Å^2)**		8,12,15,16
**B factor (CCPST*, Å^2)**		6,11,17,23

3 Fragment density observed in electron density maps

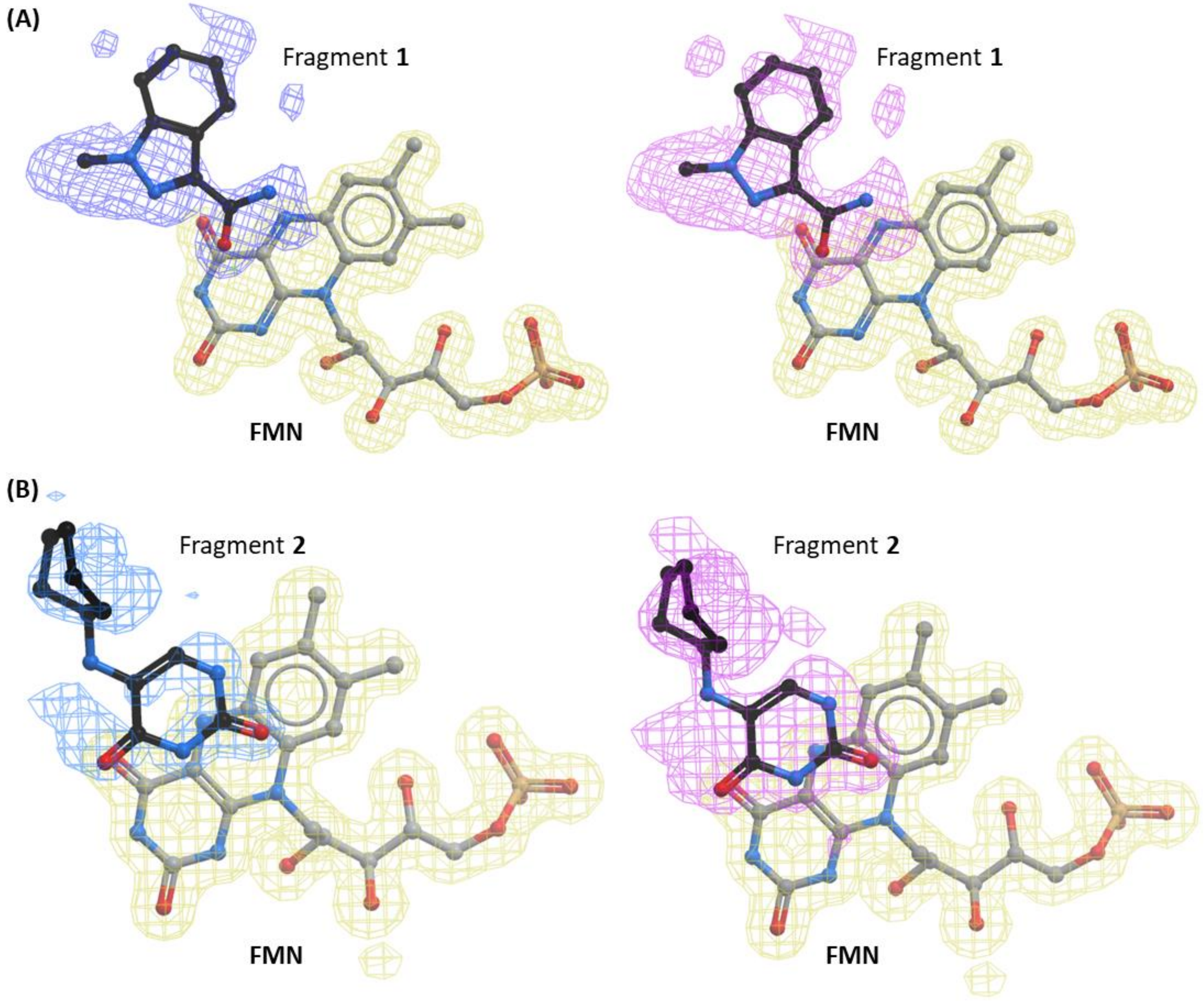

Figure S3: Comparison of ligand density observed for fragments in electron density maps obtained through either single structure refinement or the multi-crystal PanDDA method. Fragment density observed at 1σ in $2mF_o$-DF_c single crystal electron density maps (left side of each panel, blue mesh) and in $2mF_o$-DF_c multi-crystal, ground state-subtracted PanDDA electron density maps (right side of each panel, purple mesh). Density for fragments **1** – **6** are shown in panels (**A)** – (**F)**, respectively. For active site fragments (**1**, **2**, **4**, **5**), FMN density (for active site fragments 1, 2, 4 and 5) or density of residues Tyr132-Tyr134 (for gating loop fragments **3** and **6**) is also shown at 1σ to aid orientation and illustrate map quality (yellow mesh). *Continued the next page.*

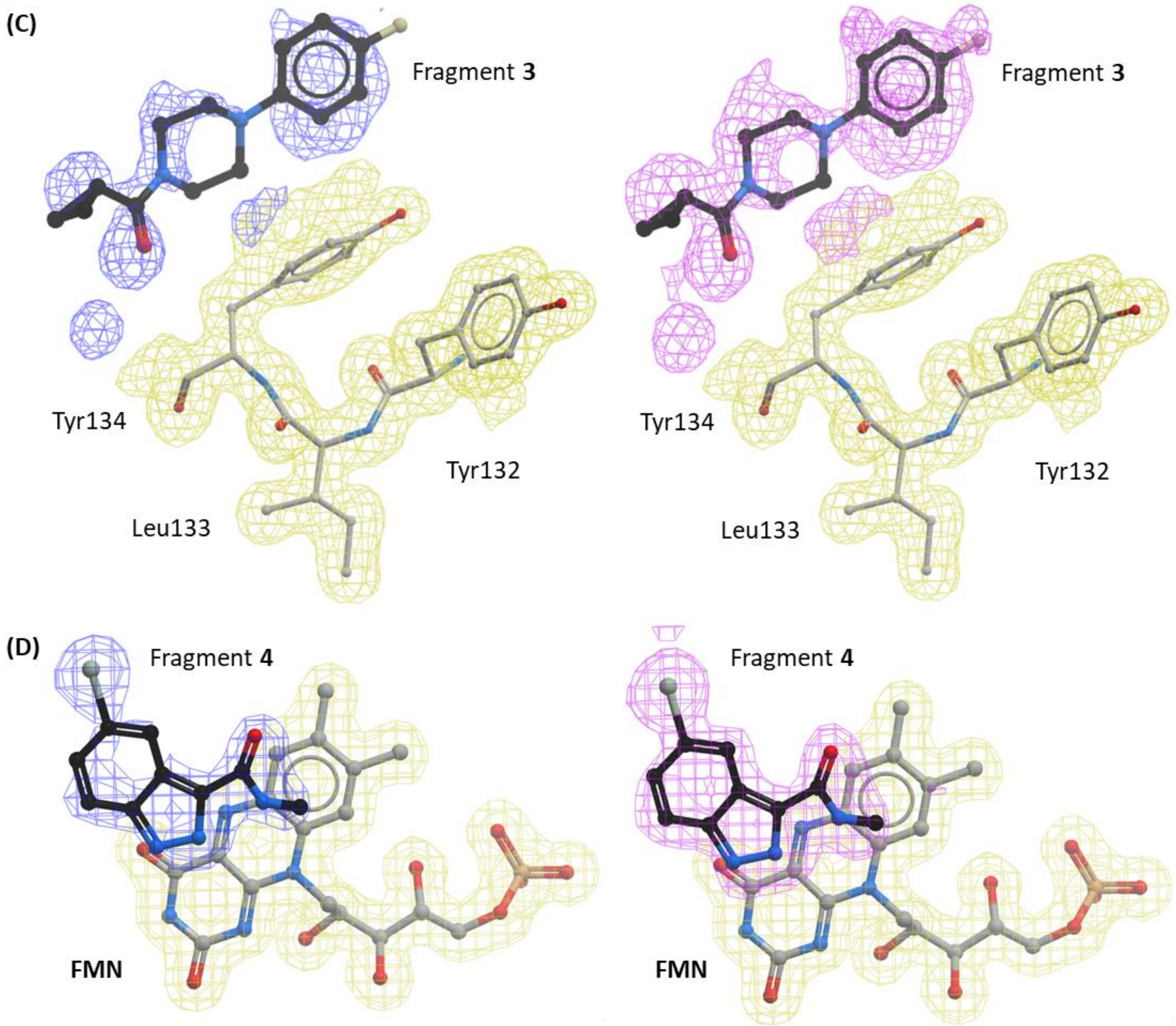

Figure S3: Comparison of ligand density observed for fragments in electron density maps obtained through either single structure refinement or the multi-crystal PanDDA method. *Continued from previous page.*

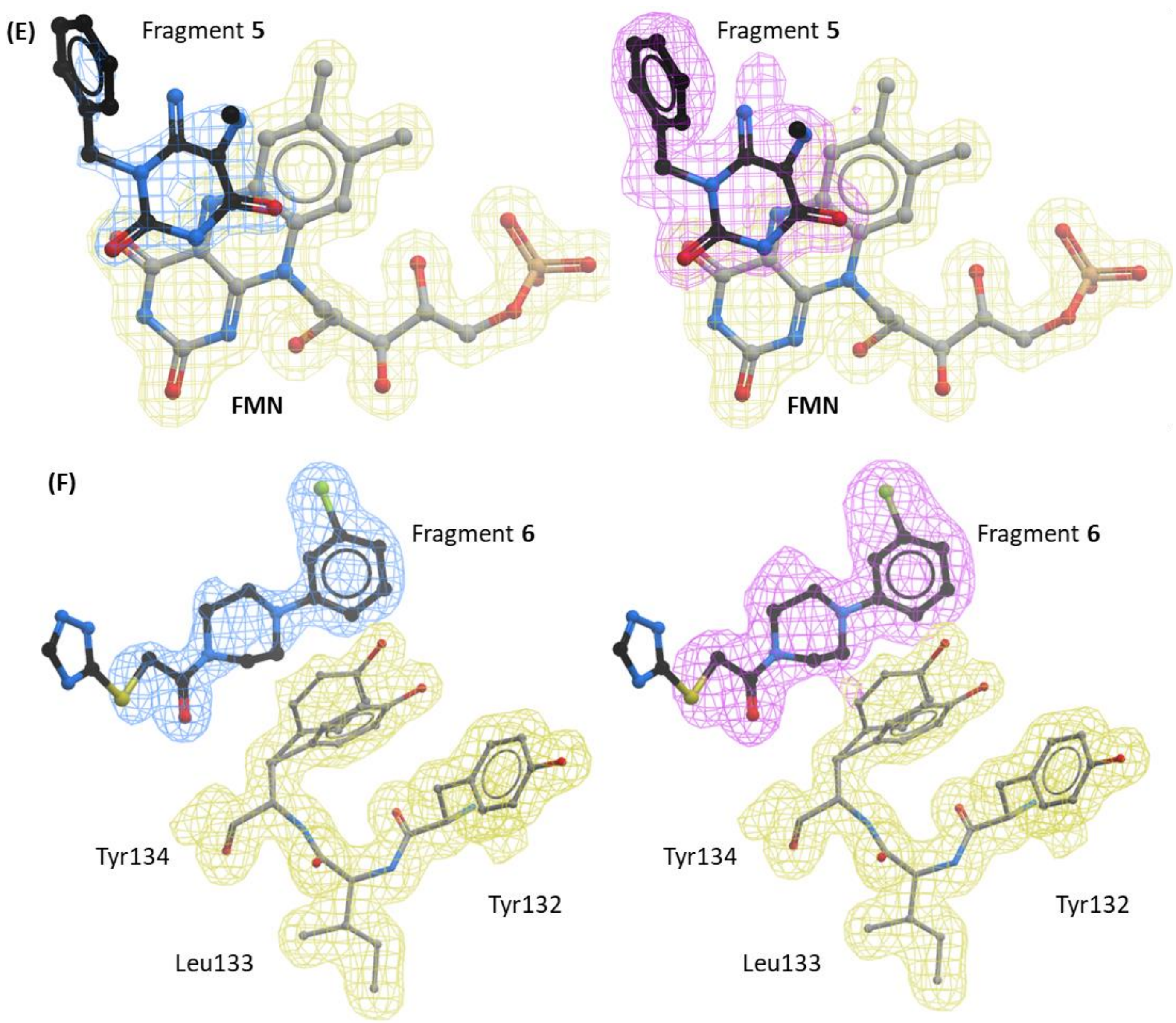

Figure S3: Comparison of ligand density observed for fragments in electron density maps obtained through either single structure refinement or the multi-crystal PanDDA method. *Continued from previous page.*

4 Co-crystallization with CCPST.

To co-crystallize hHAO1 with CCPST, 13.7 mg/mL of purified hHAO1 was pre-incubated with 2 mM CCPST (prepared as a 100 mM stock in DMSO), and crystals were grown by sitting-drop vapor diffusion at 4 °C, equilibrated against a well solution of 30% PEG1000, sodium malonate-imidazole-boric acid buffer, pH 8.0. Crystals were cryo-protected with 20-25% ethylene glycol and flash cooled in liquid nitrogen. Diffraction data were collected at Diamond Light Source beamline I04, and indexed, integrated, and scaled via the automated Xia2 pipeline (Winter 2010). Molecular replacement, using the hHAO1-FMN-glyocolate structure (PDB 6gmb) as the search template, subsequent model building, and refinement were performed in the CCP4 program suite (Winn et al. 2011).

A low-resolution crystal structure hHAO1-FMN-CCPST was previously published (PDB 2W0U, 2.8 Å resolution) (shown in yellow in Figure S3A) (Bourhis et al. 2009). We were able to extend the previously reported data by co-crystallizing our hHAO1 construct with CCPST at 1.2 Å (PDB 6gmc; Table S1, shown in blue in Figure S3). Our high-resolution structure shows CCPST, coordinated by invariant active site residues (Tyr132, Arg167, His260, Arg263) with outward rotation of the Trp110 indole sidechain to accommodate the bulky ligand and displacement of Tyr134 sidechain to avoid clashing with the displaced Trp110 (Figure S3B). Our structure is not fully occupied by CCPST and so two conformations, representing the bound and unbound state, are observed for these sidechains and no displacement of Tyr208 was observed.

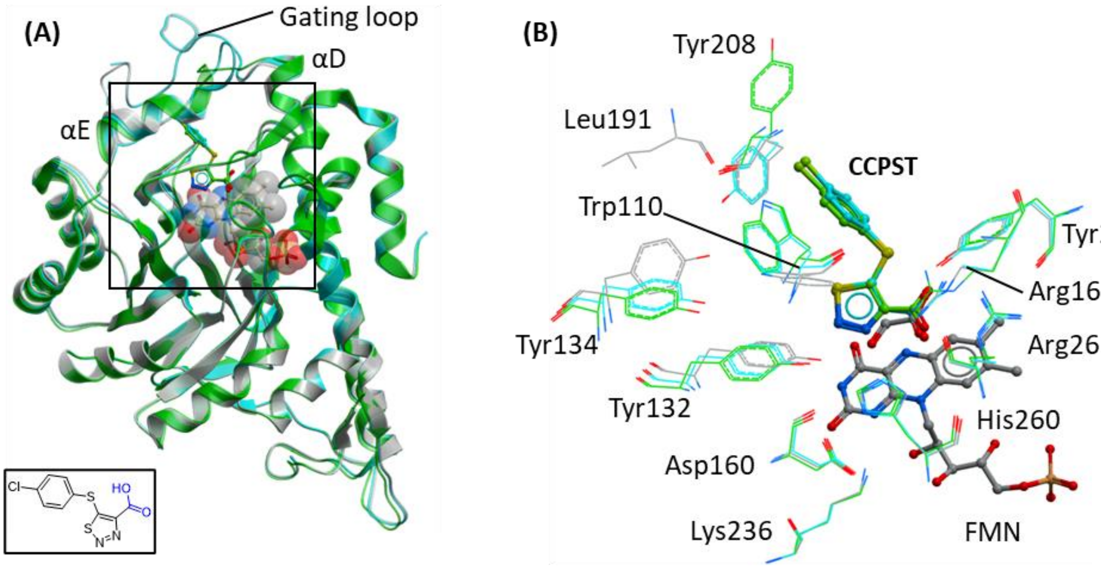

Figure S4: Structural characterization of CCPST, a known hHAO1 inhibitor. (A) Superimposed HAO1-FMN-glyoxylate structure (6gmb, grey), previously published HAO1-FMN-CCPST structure (2w0u, yellow) and HAO1-FMN-CCPST structure solved in this work (6gmc, blue). FMN is shown as spacefill and sticks whereas the other active site ligand (glycolate/ CCPST) is shown as grey, yellow and blue sticks as appropriate. *Inset*: Chemical structure of CCPST. The substrate-mimicking carboxylic acid, found in most HAO inhibitors, is highlighted in blue. **(B)** Close-up view of the boxed area shown in panel A, illustrating the binding mode of CCPST. Protein residues that interact with CCPST are shown as lines colored as in panel A. FMN, glycolate and CCPST are shown as sticks.

Table S3: Role of key hHAO1 residues in catalysis. Equivalent sGOX residue is in brackets. Where binding of an inhibitor causes changes in the observed position of a residue within the relevant co-structure, the PDB code for that co-structure is given in the right-hand column.

Residue	Residue role in catalysis	PDB IDs of structures showing changes at this position
Tyr26 (Tyr24)	Hydrogen bond with glycolate carboxylic acid	6w45, 1al8
Trp110 (Trp108)	Van der Waals with glycolate/glyoxylate; hydrogen bond with Tyr134 sidechain for gating loop conformation	All sidechains rotated 90-180°
Tyr132 (Tyr129)	Hydrogen bonds with glycolate hydroxyl/ glyoxylate carbonyl; stabilizes transition state	0.3 – 1.4 Å shift
Tyr134 (Tyr131)	Hydrogen bonds with Trp110 sidechain for gating loop conformation	0.9 – 3 Å shift
Asp160 (Asp157)	Activates His260	1al8
Arg167 (Arg164)	Hydrogen bond with glycolate carboxylic acid	6w45, 6w44
Leu191 (none)	Hydrogen bond with Tyr208 sidechain for gating loop conformation	Disordered in all structures
Tyr208 (none)	Hydrogen bond with Tyr134 sidechain and Leu191 backbone for gating loop conformation	2.8 – 10.5 Å shift
Lys236 (Lys230)	Lowers pKa of FMN N5 nitrogen to facilitate electron transfer	None
His260 (His254)	Helps transfer protons from glycolate to FMN	None
Arg263 (Arg257)	Hydrogen bond with glycolate carboxylic acid	6w45, 1al8

5 Comparison of crystal structures with known inhibitors of HAO1

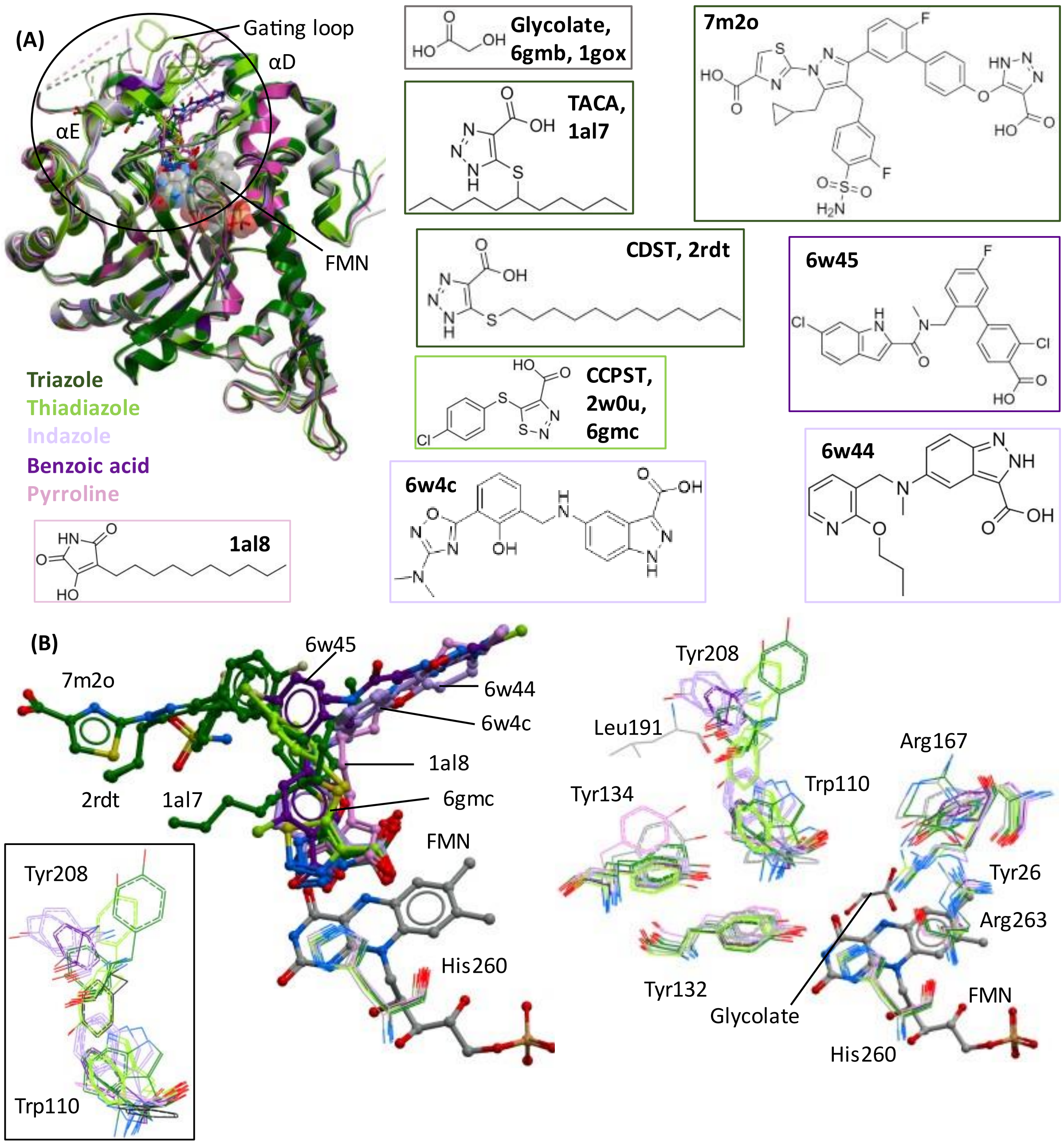

Figure S5: Comparison of binding modes of known orthosteric inhibitors of HAO1. (A) Superimposed structures of thiadiazole carboxylic acid (2w0u, 6gmc; yellow ribbon), triazole carboxylic acid (2rdt, 1al7, 7m2o; green ribbon), dioxo-pyrroline (1al8; orange ribbon), benzoic acid (6w45; purple ribbon) and indazole carboxylic acid (6w4c, 6w44; blue ribbon) inhibitors bound to either spinach glycolate oxidase (sGOX; 1al7, 1al8) or hHAO1 (other structures). **(B)** Close-up view of circled area in panel A, showing compounds as sticks (left) or active site residues as lines (right) from superimposed inhibitor-bound structures. FMN and glycolate from pdb 6gmb are shown as grey sticks. *Inset:* Observed conformations of Trp110 and Tyr208, across superimposed inhibitor-bound structures, taken from panel B. *Continued next page.*

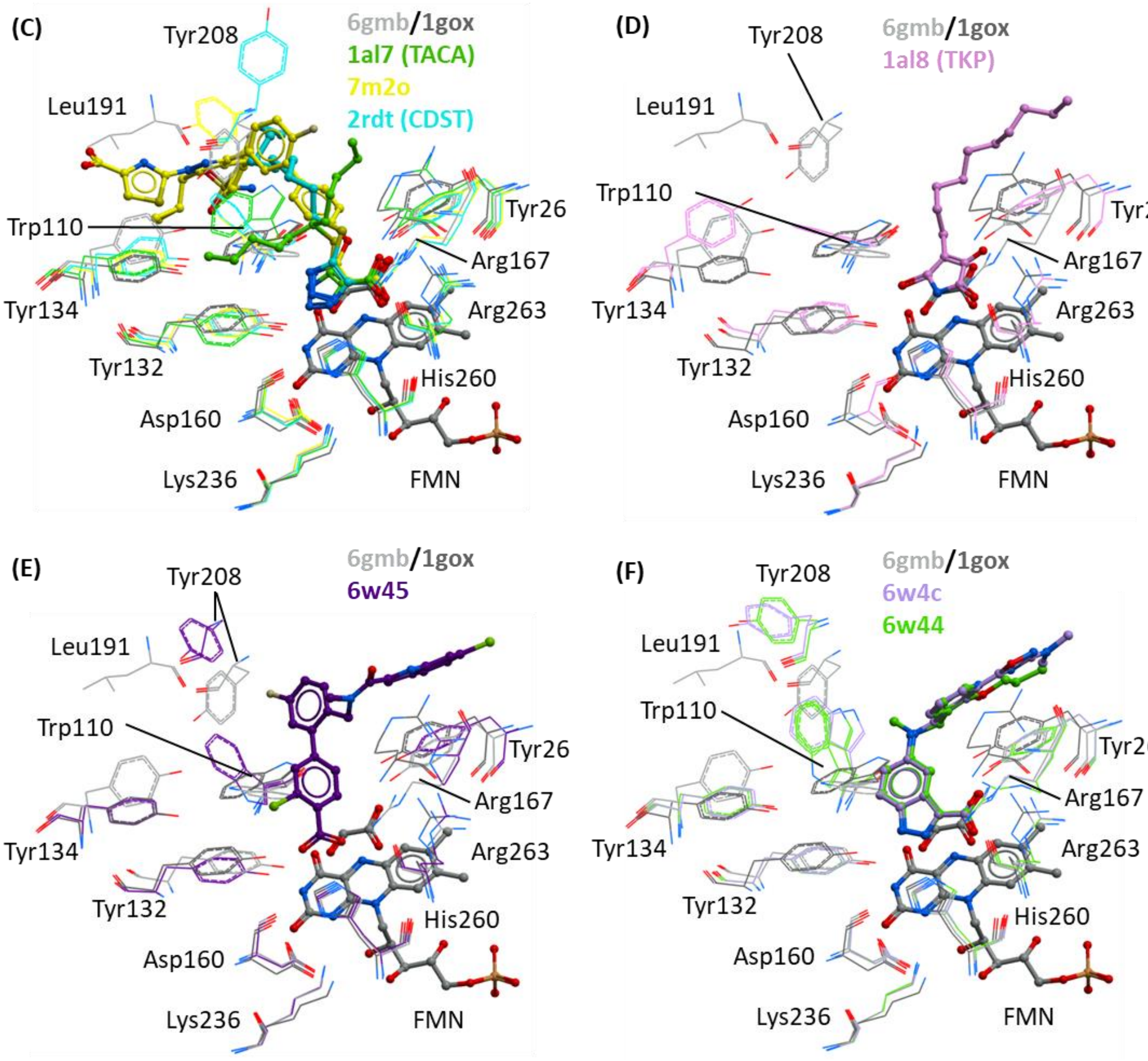

Figure S5: Comparison of binding modes of known orthosteric inhibitors of HAO1. *Continued.* **(C-F)** Close-up view of boxed area in panel A, showing active site residues conformations of HAO1 when bound to inhibitors containing triazole carboxylic acid (C), dioxo-pyrroline (D), benzoic acid (E) or indazole carboxylic acid (F). Protein residues are shown as lines and inhibitors are shown as sticks, both colored according to the inset key. Glycolate-bound hHAO1 and glyoxylate-bound sGOX active site residues are shown in light and dark grey lines respectively. FMN and glycolate are shown as grey sticks. Equivalent close-up view for thiadiazole carboxylic acid inhibitor-bound structures (CCPST; 2w0u, 6gmc) are shown in Figure S4B.

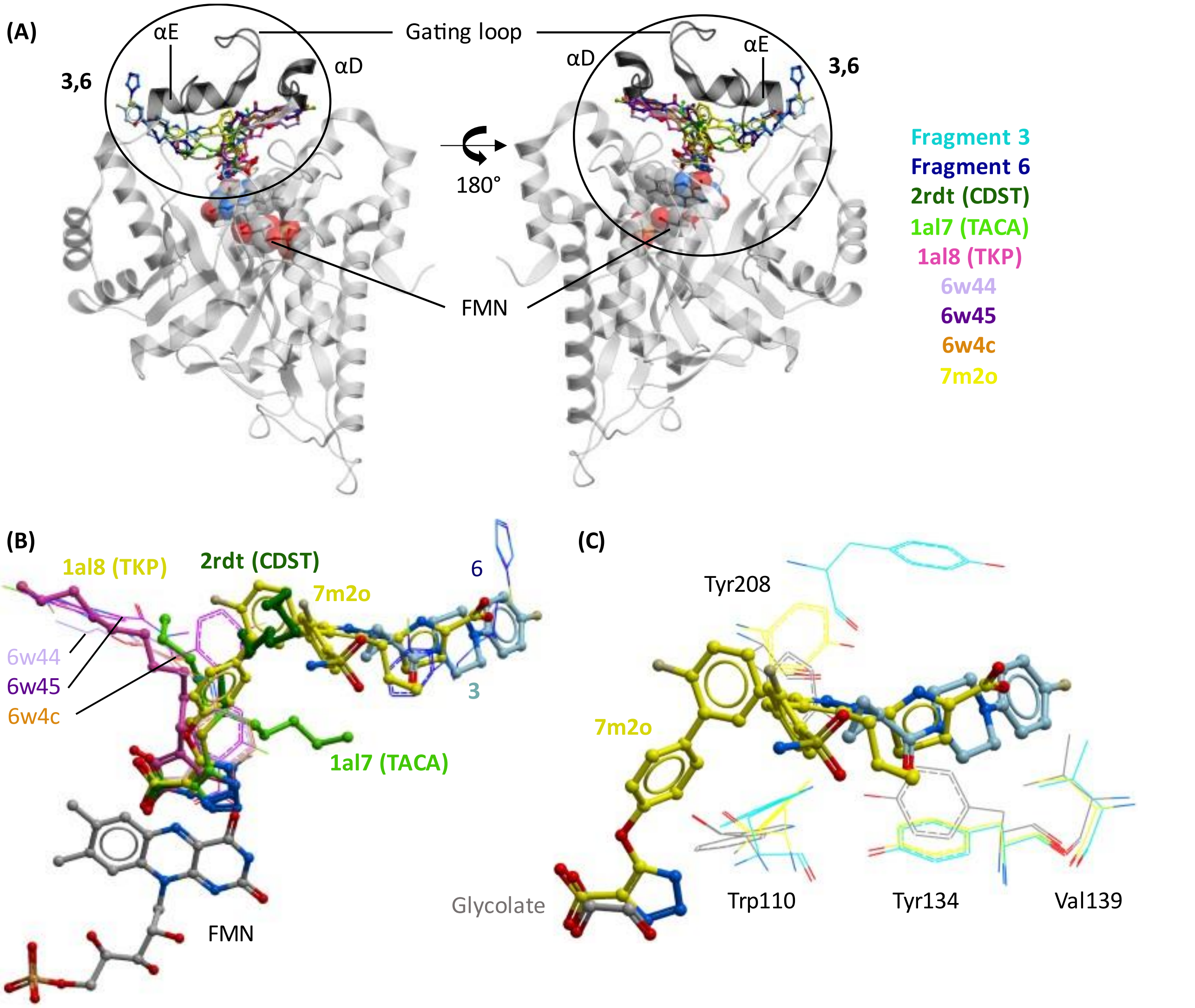

Figure S6: Comparison of binding mode of known orthosteric inhibitors of HAO1 with gating loop site fragments identified in this work. (A) Comparison of the binding mode of the gating loop fragments (**3**, **6**) and the seven HAO1 inhibitors (PDB 2rdt, 1al7, 1al8, 6e44, 6w45, 6w4c, 7m2o) that are large enough to reach the gating loop pocket shown as sticks on the glycolate-bound structure (6gmb). Ligand sticks are colored according to the inset key. FMN is shown as sticks and spacefill. The gating loop is colored black. **(B)** Close-up view of circled area in the right-hand structure of panel A, showing the relative binding mode of gating loop fragments and selected orthosteric HAO1 inhibitors. Four orthosteric inhibitors, each exiting the active site in a different direction, are shown a sticks, as is fragment 3. The three remaining orthosteric inhibitors and fragment 6 occupy binding pockets represented by other compounds and so are shown as lines. Both are colored according to the key in panel A. **(C)** Close-up view of circled area in left-hand structure of panel A, highlighting key interacting residues of the gating loop pocket for superimposed structures with gating loop fragments and the orthosteric inhibitor that reaches the gating loop pocket. Protein residues are shown as lines and ligands are shown as sticks, both colored according to the key in panel A.

6 Establishing reagents and crystal system for hHAO1 inhibitor development

6.1 Validating HAO1 binding assay (surface plasmon resonance) using CCPST

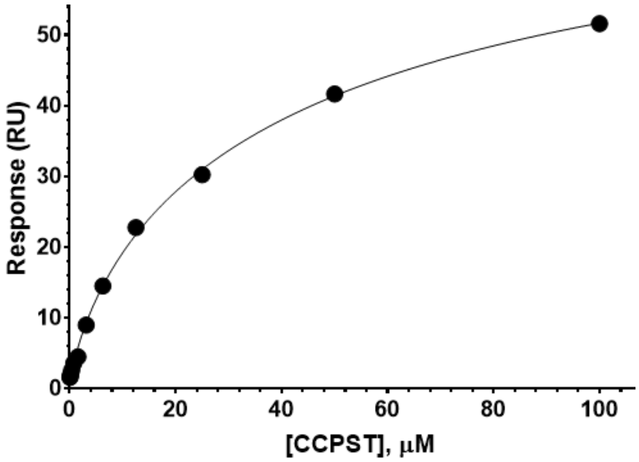

Figure S7: Validation of hHAO1 binding assay using known inhibitor CCPST. Characterisation of CCPST (0-100 μM) binding to hHAO1 (immobilised to 5000 RU on Ni-NTA chip) by surface plasmon resonance. Plot of relative response, in response units (RU), against CCPST concentration, to determine K_D for hHAO1. Data are for an n=1 experiment.

6.2 Establishing parameters for Amplex Red activity assay

HAO1 activity was measured by coupling production of H_2O_2 by hHAO1 with the H_2O_2-dependent conversion of Amplex Red to its fluorescent product resorufin by hydrogen peroxidase (Figure S7A). The relationship between fluorescence signal and hydrogen peroxide concentration was linear up to 75 μM hydrogen peroxide (R^2 = 0.995; Figure S7B). To determine an incubation period resulting in measurements being made at initial rate conditions that also generated sufficient fluorescence signal, activity of 30 nM hHAO1 in the presence of 30 μM glycolate was measured every 1.5 minutes for a period of 60 minutes (Figure S7C). From this experiment, the optimal incubation period was determined to be 10 minutes. To confirm recombinant hHAO1 used in this work was active, the reaction rate of 30 nM hHAO1 was measured across a range of glycolate (Figure S7C) and 2-hydroxypalmitate (Figure S7D) concentrations. The same maximal velocity ($Vmax^{apparent}$ = 1.5 μM H_2O_2/minute) was reached at high concentrations of both substrates, albeit requiring an order of magnitude greater concentration of 2-hydroxypalmitate than of glycolate. The calculated $Km^{apparent}$ glycolate was 18.9 ± 0.8 μM (Figure S7C), which is fairly similar to the published value of 74.5 μM (Wang et al. 2016). In agreement with published work (Jones, Morrell, and Gould 2000), hHAO1 was much less active against 2-hydroxypalmitate with a calculated $Km^{apparent}$ of 1.2 ± 0.4 mM (Figure S7D).

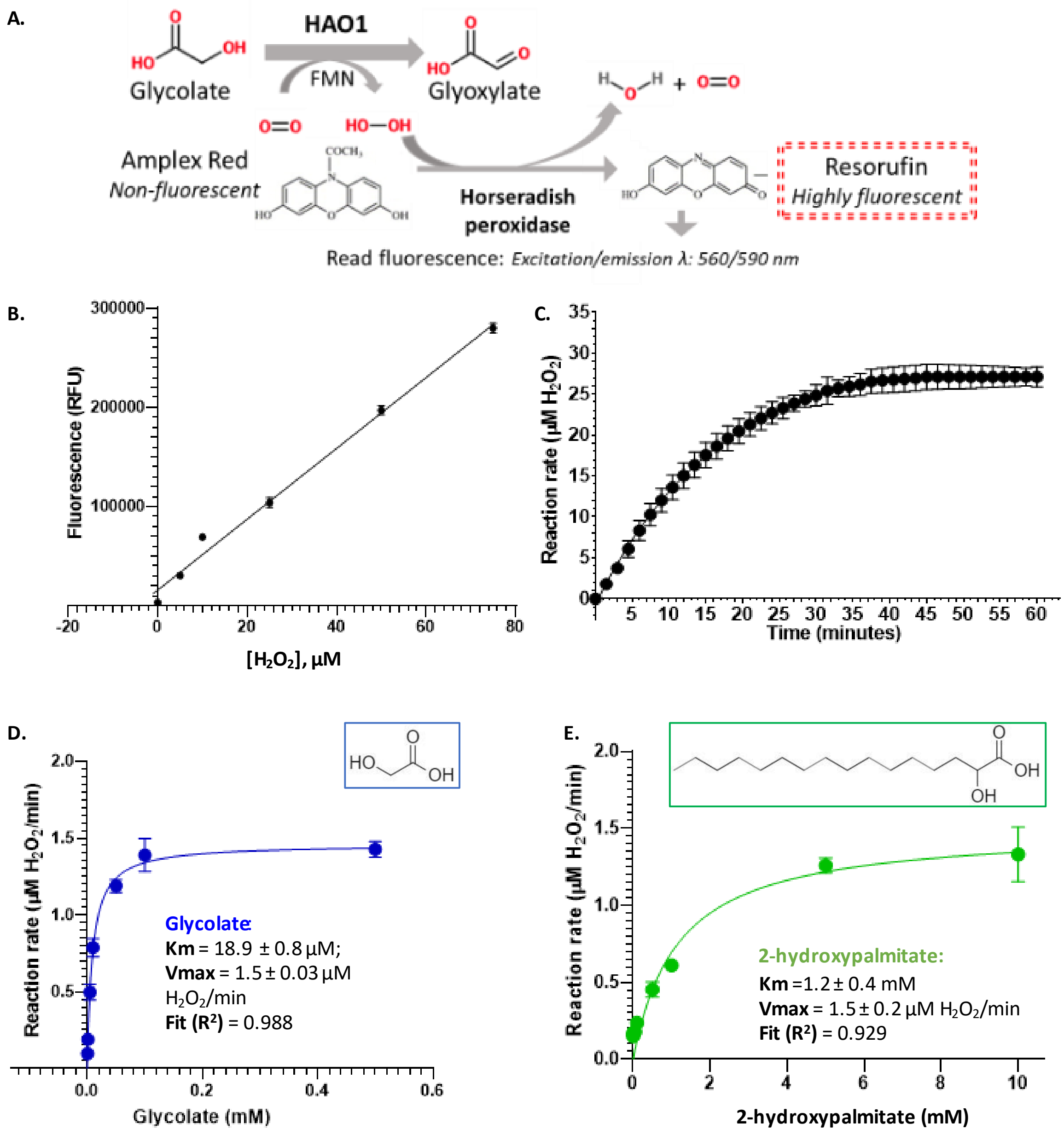

Figure S8: Set-up of hHAO1 Amplex Red activity assay. (A) Schematic showing how hHAO1 activity is measured using the Amplex Red assay. **(B)** Plot of reaction rate (hydrogen peroxide consumed, µM) of 30 nM hHAO1 over time (minutes) in the presence of 30 µM glycolate. **(C)** Plot of relative fluorescence versus hydrogen peroxide concentration showing the standard curve fitted within the linear range of the Amplex Red reagent. **(D)** Michaelis-Menten plot of glycolate concentration versus reaction rate for 30 nM hHAO1. *Inset*: chemical structure of glycolate. **(E)** Michaelis-Menten plot of 2-hydroxypalmitate concentration versus reaction rate for 30 nM hHAO1. *Inset*: chemical structure of 2-hydroxypalmitate.

6.3 Validating activity assay using known inhibitor CCPST

To test our protein preparation and assay set-up, we purchased 4-carboxy-5-[(4-chlorophenyl)sulfanyl]-1,2,3-thiadiazole (CCPST), a known low-µM potency inhibitor of hHAO1 for

which a low-resolution crystal structure was previously published (PDB 2W0U, 2.8 Å resolution) (Bourhis et al. 2009), and measured its inhibition of hHAO1 (Figure S8).

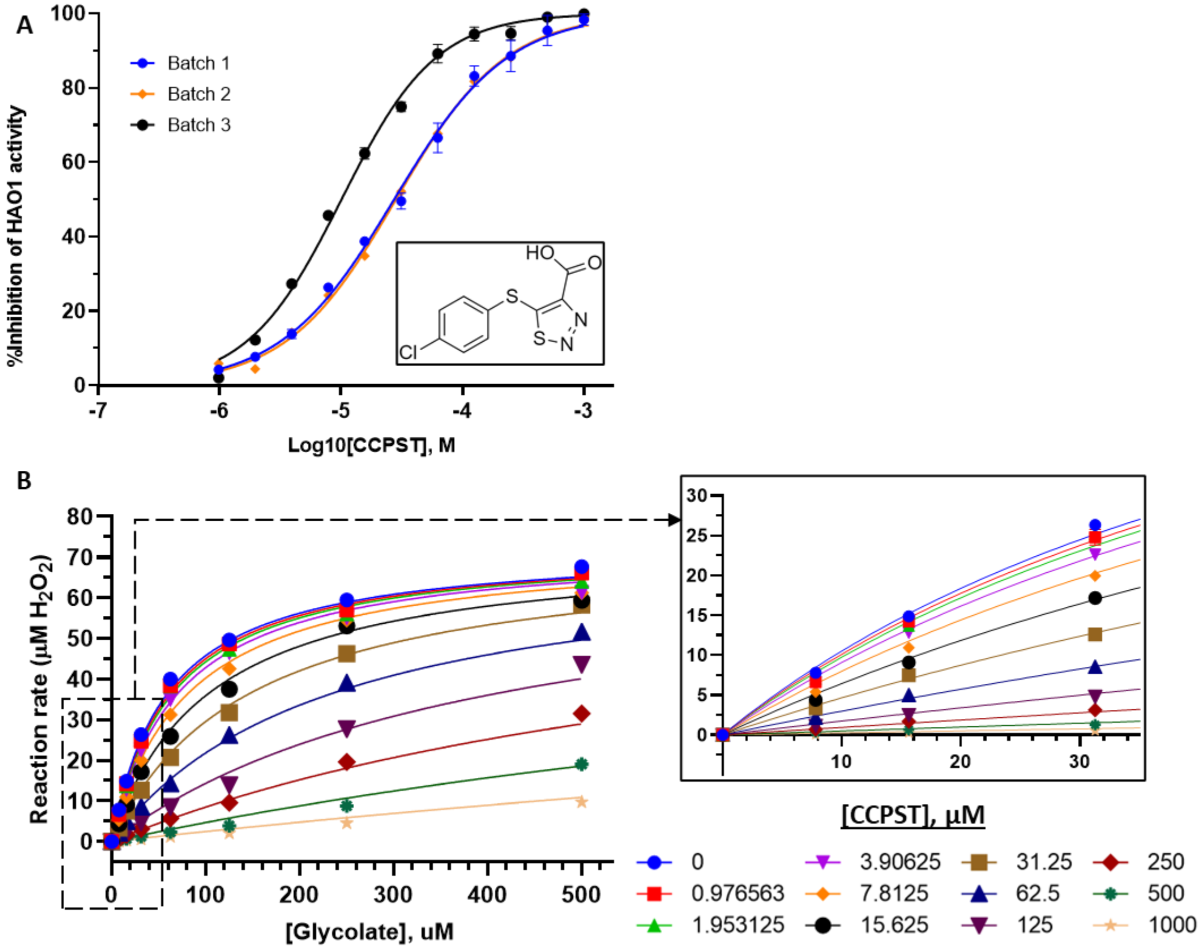

Figure S9: Validation of hHAO1 activity assay using known inhibitor CCPST. (A) Concentration-response curve for CCPST inhibition of three preparations of hHAO1 measured in the Amplex Red activity assay at 30 µM glycolate. Error bars are standard deviation of three replicates. *Inset*: Chemical structure of CCPST. **(B)** Least-squares non-linear fit of HAO1 reaction rate (total hydrogen peroxide produced after 15 minutes reaction, µM) against increasing glycolate concentrations (0 -500 µM) in the presence of different concentrations of CCPST (0 – 1 mM). Curves were fitted to competitive inhibition model, the best fitting Enzyme kinetics – Inhibition equation, in GraphPad Prism. Error bars are standard deviation of three replicates. *Inset*: Close-up view of plot showing HAO1 reaction rate (total hydrogen peroxide produced after 15 minutes reaction, µM) against increasing glycolate concentrations (0 -31.25 µM) in the presence of different concentrations of CCPST (0 – 1 mM).

HAO inhibition by CCPST has previously been reported for purified mGO (IC_{50} 43-198 µM (Martin-Higueras, Luis-Lima, and Salido 2016; Moya-Garzón et al. 2018)), rat LCHAO (equivalent to hHAO2) (IC_{50} 3.6 µM (Chen et al. 2012)), flavin dehydrogenase domain of yeast flavocytochrome b2 (IC_{50} 6 µM (Chen et al. 2012)) and hHAO1 (IC_{50} 4.5 µM (Chen et al. 2012)). However, puzzlingly, published kinetics of CCPST inhibition against these targets report non-competitive inhibition mode (Chen et al. 2012; Martin-Higueras, Luis-Lima, and Salido 2016), which would indicate non-orthosteric binding, contradicting the active site binding observed in the three published HAO-CCPST structures (3sgz, 2w0u, 6gmc). Inhibition of hHAO1 by CCPST observed in this work was consistent with these published values, with an average IC_{50} value of 22 ± 9 µM across three preparations of hHAO1 (Figure

S8A). In this work, however, we observed a competitive inhibition mode (Figure S8B), consistent with the orthosteric binding expected from the crystal structures.

7 Inhibition mode determination for active site follow-up compounds 7, 9-12

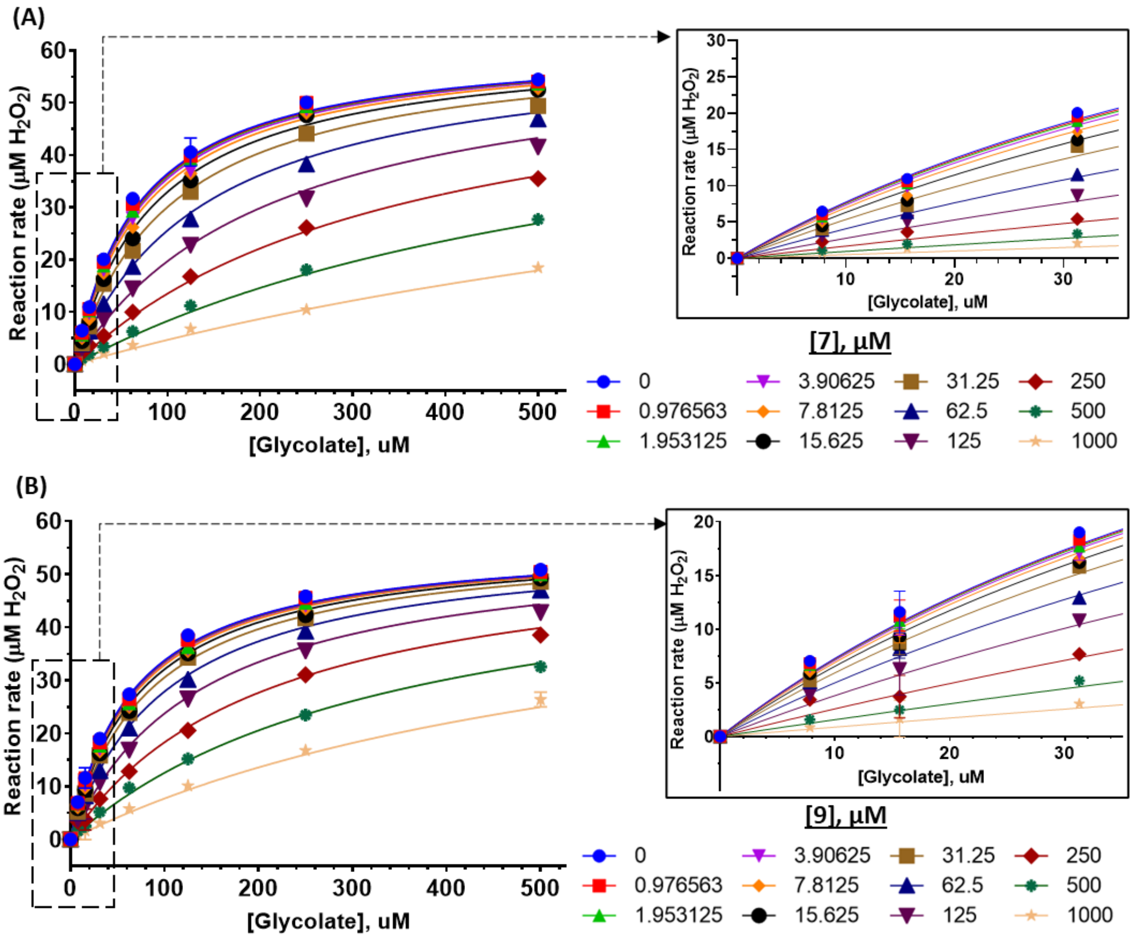

Figure S10: Inhibition mode of active site follow-up compounds with respect to glycolate. (**A-E**) Least-squares non-linear fit of HAO1 reaction rate (total H_2O_2 produced after 15 minutes reaction, µM) against increasing glycolate concentrations (0 -500 µM) in the presence of different concentrations of compounds **7**, **9-12** (0 – 1 mM; panels A-E respectively). Curves were fitted to competitive inhibition model, the best fitting Enzyme kinetics – Inhibition equation, in GraphPad Prism. Error bars are standard deviation of three replicates. *Inset*: Close-up view of plot showing HAO1 reaction rate (total H_2O_2 produced after 15 minutes reaction, µM) against increasing glycolate concentrations (0 -35 µM) in the presence of different concentrations of compounds 7, 9-12 (0 – 1 mM; panels A-E respectively). *Continued next page.*

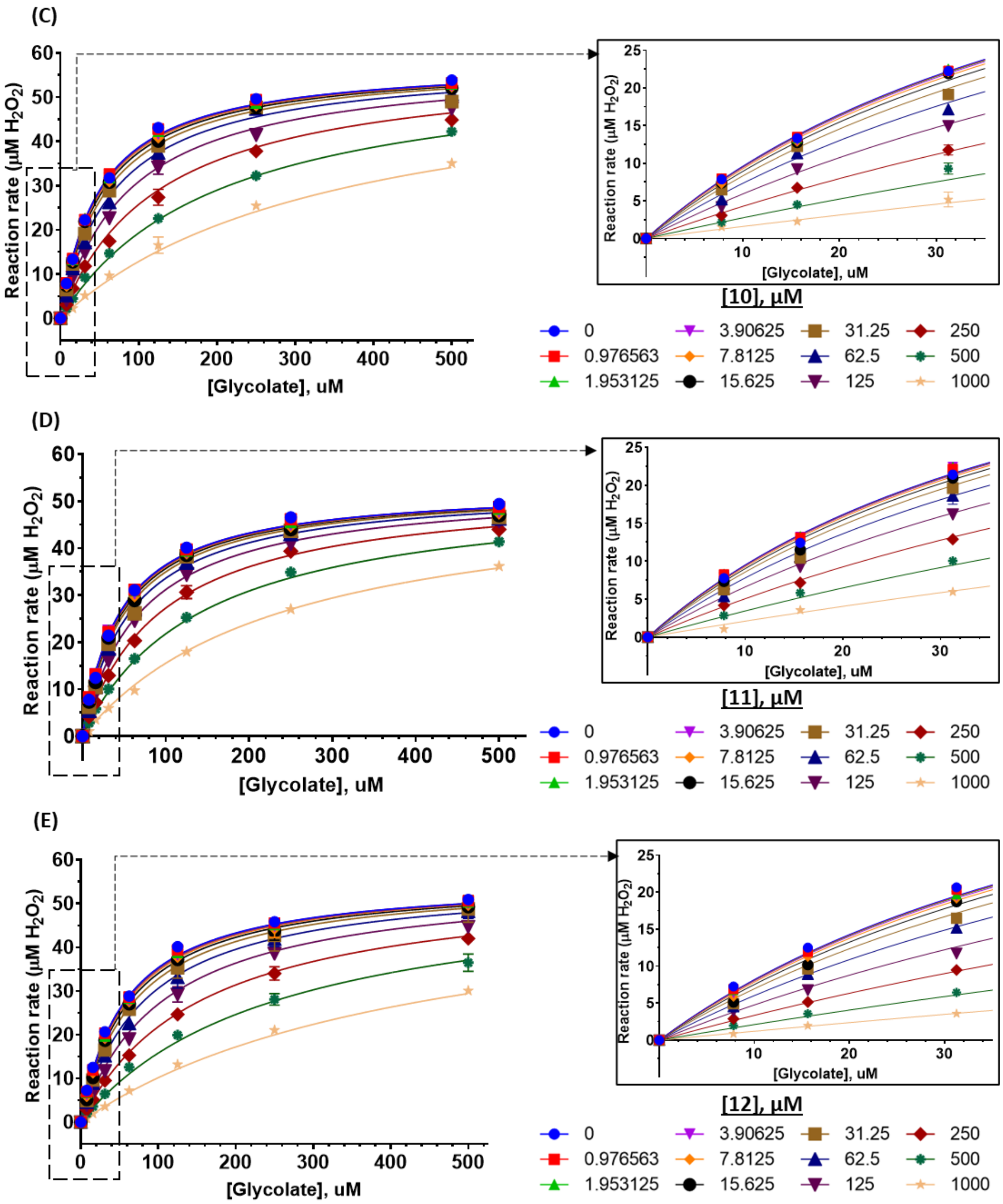

Figure S10: Inhibition mode of active site follow-up compounds with respect to glycolate. *Continued from previous page.*

8 References

Bourhis, J.M., Vignaud, C., Pietrancosta, N., Guéritte, F., Guénard, D., Lederer, F. and Lindqvist, Y. (2009). Structure of Human Glycolate Oxidase in Complex with the Inhibitor 4-Carboxy-5-[(4-Chlorophenyl)Sulfanyl]-1,2,3-Thiadiazole. *Acta Crystallographica Section F: Structural Biology and Crystallization Communications* 65 (12): 1246–53. doi:10.1107/S1744309109041670

Chen, Z.W., Vignaud, C., Jaafar, A., Lévy, B., Guéritte, F., Guénard, D. et al. (2012). High Resolution Crystal Structure of Rat Long Chain Hydroxy Acid Oxidase in Complex with the Inhibitor 4-Carboxy-5-[(4-Chlorophenyl)Sulfanyl]-1, 2, 3-Thiadiazole. Implications for Inhibitor Specificity and Drug Design. *Biochimie* 94 (5): 1172–79. doi:10.1016/j.biochi.2012.02.003

Corpet, F. (1988). Multiple Sequence Alignment with Hierarchical Clustering. *Nucleic Acids Research* 16 (22): 10881–90. doi:10.1093/nar/16.22.10881

Jones, J.M., Morrell, J.C. and Gould, S.J. (2000). Identification and Characterization of HAOX1, HAOX2, and HAOX3, Three Human Peroxisomal 2-Hydroxy Acid Oxidases. *Journal of Biological Chemistry* 275 (17): 12590–97. doi:10.1074/jbc.275.17.12590

Martin-Higueras, C., Luis-Lima, S. and Salido, E. (2016). Glycolate Oxidase Is a Safe and Efficient Target for Substrate Reduction Therapy in a Mouse Model of Primary Hyperoxaluria Type I. *Molecular Therapy* 24 (4): 719–25. doi:10.1038/mt.2015.224

Moya-Garzón, M.D, Martín-Higueras, C., Peñalver, P., Romera, M., Fernandes, M.X., Franco-Montalbán, F. et al. (2018). Salicylic Acid Derivatives Inhibit Oxalate Production in Mouse Hepatocytes with Primary Hyperoxaluria Type 1. *Journal of Medicinal Chemistry* 61 (16): 7144–67. doi:10.1021/acs.jmedchem.8b00399

Robert, X. and Gouet, P. (2014). Deciphering Key Features in Protein Structures with the New ENDscript Server. *Nucleic Acids Research* 42 (W1). doi:10.1093/nar/gku316

Wang, M., Xu, M., Long, Y., Fargue, S., Southall, N., Xin Hu, X. et al. 2016. High Throughput Cell-Based Assay for Identification of Glycolate Oxidase Inhibitors as a Potential Treatment for Primary Hyperoxaluria Type 1. *Scientific Reports* 6 (1): 34060. doi:10.1038/srep34060.

Winn, M.D, Ballard, C.C., Cowtan, K.D., Dodson, E.J., Emsley, P., Evans, P.R. et al. (2011). Overview of the CCP4 Suite and Current Developments. *Acta Crystallographica Section D* 67 (4): 235–42. doi:10.1107/S0907444910045749.

Winter, G. (2010). Xia2: An Expert System for Macromolecular Crystallography Data Reduction. *Journal of Applied Crystallography* 43 (1): 186–90. doi:10.1107/S0021889809045701.
